# Effects of *ANRIL* variants on the risk of ischemic stroke: a meta-analysis

**DOI:** 10.1042/BSR20182127

**Published:** 2019-05-15

**Authors:** Cheng Tan, Junzhi Liu, Jun Wei, Shoujun Yang

**Affiliations:** 1 Department of Neurology, China-Japan Union Hospital of Jilin University, Changchun 130033, China; 2 Department of Quality Control, China-Japan Union Hospital of Jilin University, Changchun 130033, China; 3 Department of Neurosurgery, China-Japan Union Hospital of Jilin University, Changchun 130033, China

**Keywords:** Antisense non-coding RNA in the INK4 locus (ANRIL), Gene variants, Ischemic stroke (IS), Meta-analysis

## Abstract

***Background*** *:* Several studies investigated the relationship between antisense non-coding RNA in the INK4 locus (*ANRIL*) variants and the risk of ischemic stroke (IS), yet whether *ANRIL* variants are associated with IS remain controversial. Therefore, we performed the present study to obtain a more conclusive result. ***Methods:*** Literature retrieval was conducted in PubMed, Medline and Embase. Odds ratios (ORs) and 95% confidence intervals (CIs) were calculated. ***Results:*** Eighteen studies were enrolled for analyses. Pooled overall analyses showed that rs2383206 (recessive model: *P*=0.002, OR = 1.22, 95%CI 1.08–1.38; allele model: *P*=0.003, OR = 0.90, 95%CI 0.84–0.96) and rs10757274 (allele model: *P=*0.006, OR = 0.91, 95%CI 0.86–0.97) variants were significantly associated with an increased risk of IS. Further subgroup analyses by ethnicity revealed that rs2383206, rs10757274 and rs10757278 variants were all significantly correlated with an increased risk of IS in Asians. Additionally, rs10757278 polymorphism was also significantly correlated with an increased risk of IS in Caucasians. ***Conclusions:*** Our findings indicated that rs2383206, rs10757274 and rs10757278 variants may impact individual susceptibility to IS in Asians. Moreover, rs10757278 polymorphism may also impact individual susceptibility to IS in Caucasians.

## Introduction

Ischemic stroke (IS) is one of the leading causes of morbidity and mortality all over the world [1]. So far, the exact cause of IS remains ambiguous in spite of extensive investigations. Nevertheless, accumulating evidence suggests that genetic factors may play crucial parts in its pathogenesis. First, numerous genetic variants were found to be associated with an increased risk of IS by previous genetic association studies [2–4]. Second, screening of common causal variants was also proved to be a cost-efficient way to predict the individual risk of developing IS [5,6]. Overall, these findings supported that genetic predisposition is crucial for the occurrence and development of IS.

Antisense non-coding RNA in the INK4 locus (*ANRIL*) is located on human chromosome 9p21, a region that has been repeatedly linked to atherosclerosis and its associated ischemic vascular diseases [7]. Previous studies demonstrated that the expression levels of several neighbor protein-encoding genes like cyclin-dependent kinase inhibitors 2A (*CDKN2A*), *CDKN2B* and methylthioadenosine phosphorylase (*MTAP*) are modulated by *ANRIL*. It was shown that the above-mentioned proteins were abundantly expressed in atherosclerotic lesions, and they could promote atherosclerosis by impacting vascular remodeling, thrombogenesis and plaque stability [8]. Additional, recent experimental analyses also showed that *ANRIL* could promote inflammation by inhibiting caspase recruitment domain family member (CARD) 8 and activating the NF-κB pathway [9]. Considering the critical role of *ANRIL* in regulating atherosclerosis and inflammation as well as the close relationship between these two processes and IS, it is believed that functional *ANRIL* variants may also be involved in the development of IS.

In the past decade, several studies have already investigated potential correlations between *ANRIL* variants and the risk of IS, yet the results of these studies were controversial [10,11]. Thus, we performed the present meta-analysis to better evaluate the roles of *ANRIL* variants in IS.

## Materials and methods

### Literature search and inclusion criteria

This meta-analysis followed the Preferred Reporting Items for Systematic Reviews and Meta-analyses (PRISMA) guideline [12]. The authors conducted a systematic search of PubMed, Medline and Embase to identify potentially related literatures published up to October 2018 using the following searching strategy: (antisense noncoding RNA in the INK4 locus OR CDKN2B antisense RNA OR ANRIL OR CDKN2B-AS long non-coding RNA) AND (polymorphism OR variant OR mutation OR genotype OR allele) AND (ischemic stroke OR cerebral infarction OR brain infarction OR cerebrovascular disease). Furthermore, the references of retrieved articles were also screened for other potentially relative studies.

To test the research hypothesis of this meta-analysis, included studies must meet all the following criteria: (a) case–control study on correlation between *ANRIL* variants and IS; (b) it provides genotypic and/or allelic frequency of *ANRIL* variants in cases and controls; (c) full text in English or Chinese available. Studies were excluded if one of the following criteria was fulfilled: (a) not related to *ANRIL* variants and IS; (b) case reports or case series; (c) abstracts, reviews, comments, letters and conference presentations. For duplicate reports, we only included the study with the largest sample size for analyses.

### Data extraction and quality assessment

The following data were extracted from included studies: (i) name of the first author; (ii) publication year; (iii) country and ethnicity; (iv) sample size; and (v) genotypic distribution of *ANRIL* variants in cases and controls. Additionally, the probability value (*P*-value) of Hardy–Weinberg equilibrium (HWE) was also calculated. When necessary, we wrote to the corresponding authors for raw data. We used the Newcastle–Ottawa scale (NOS) to evaluate the quality of eligible studies [13]. This scale has a score range of zero to nine, and studies with a score of more than seven were thought to be of high quality. Two reviewers conducted data extraction and quality assessment independently. Any disagreement between two reviewers was solved by discussion until a consensus was reached.

### Statistical analyses

All statistical analyses were achieved using Review Manager Version 5.3.3 (The Cochrane Collaboration, Software Update, Oxford, United Kingdom). Odds ratios (ORs) and 95% confidence intervals (CIs) were calculated to estimate strength of associations, and *P*-values ≤0.05 were considered to be statistically significant. Between-study heterogeneities were evaluated with *I^2^* statistic. Random-effect models (REMs) would be used to pool the data if *I^2^* ≥ 50%. Otherwise, fixed-effect models (FEMs) would be employed for synthetic analyses. Subgroup analyses by ethnicity were subsequently performed. Sensitivity analyses were executed to test the stability of synthetic results. Funnel plots were used to assess publication biases.

## Results

### Characteristics of included studies

**Figure 1:**
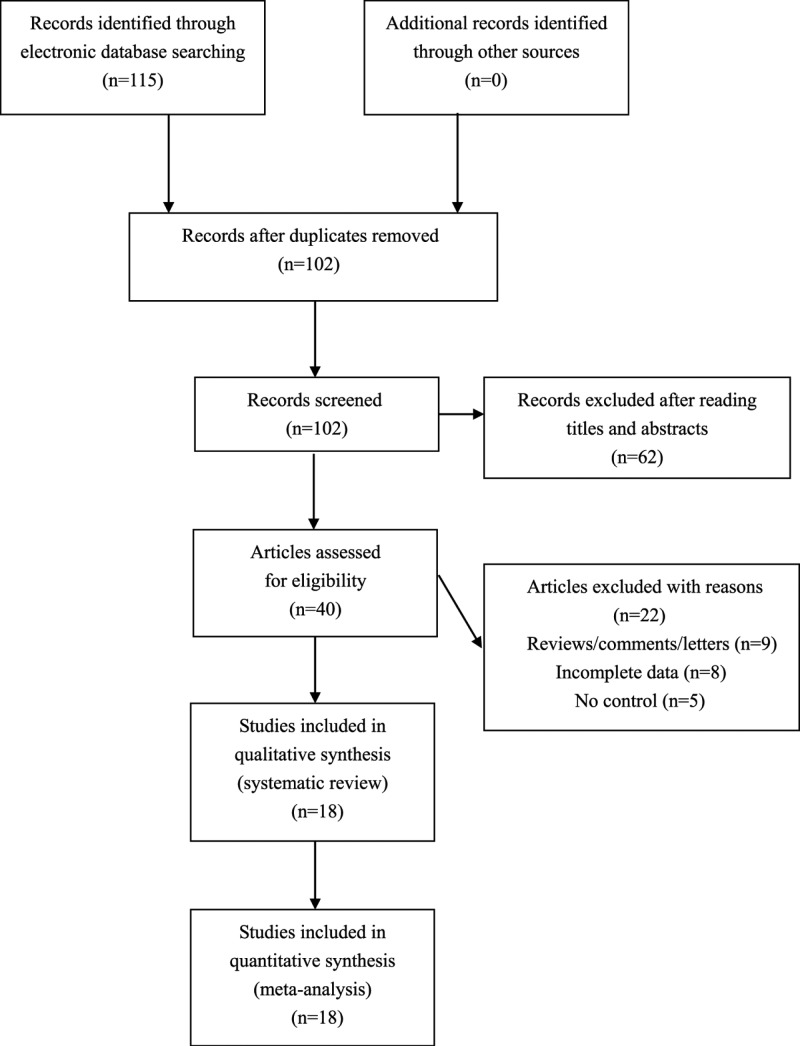
Flowchart of study selection for the present study

We found 115 potential relative articles. Among these articles, a total of 18 eligible studies which met our inclusion criteria were included for synthetic analyses (see Figure 1). The NOS score of eligible articles ranged from 7 to 8, which indicated that all included studies were of high quality. Baseline characteristics of included studies were shown in Table 1 [10,14–28].

**Table 1:** The characteristics of included studies for *ANRIL* variants and IS

First author (year)	Country	Ethnicity	Sample size	Genotype distribution		Minor allele (%) Case/Control	*P*-value for HWE	NOS score
			Case/Control	Cases	Controls			
**rs1333040**	TT/TC/CC							
Akinyemi (2018) [10]	Nigeria	African	82/247	NA	NA	42.1%/42.9%	NA	7
Cao (2016) [15]	China	Asian	569/541	267/247/55	256/225/60	31.4%/31.9%	0.323	8
Heckman (2013) [19]	U.S.A.	Mixed	264/373	93/127/44	130/179/64	40.7%/41.2%	0.859	7
Lin (2011) [23]	Taiwan	Asian	634/1352	324/259/51	649/573/130	28.5%/30.8%	0.829	7
Olsson (2011) [25]	Sweden	Caucasian	803/641	248/392/163	177/314/150	44.7%/47.9%	0.639	8
Xiong (2018) [26]	China	Asian	200/205	104/79/17	112/76/17	28.3%/26.8%	0.425	8
**rs1333049**	GG/GC/CC							
Haslacher (2016) [18]	Austria	Caucasian	151/773	33/84/34	218/374/183	50.3%/47.6%	0.402	7
Lin (2011) [23]	Taiwan	Asian	642/1361	176/313/153	395/655/311	48.2%/46.9%	0.213	7
Xiong (2018) [26]	China	Asian	200/205	53/96/51	56/102/47	49.5%/47.8%	0.966	8
Yang (2018) [28]	China	Asian	550/549	162/266/122	176/273/100	46.4%/43.1%	0.743	8
**rs2383206**	AA/AG/GG							
Ding (2009) [16]	China	Asian	991/1054	275/463/253	314/526/214	48.9%/45.3%	0.816	8
Hu (2009) [21]	China	Asian	352/423	97/188/67	154/191/78	45.7%/41.0%	0.169	7
Xiong (2018) [26]	China	Asian	200/205	56/96/48	61/98/46	48.0%/46.3%	0.579	8
Zee (2007) [29]	U.S.A.	Mixed	254/254	NA	NA	46.6%/46.3%	NA	7
Zhang (2012) [30]	China	Asian	1190/1664	359/569/262	514/833/317	45.9%/44.1%	0.529	8
**rs2383207**	GG/GA/AA							
Gschwendtner (2009) [17]	Germany	Caucasian	962/4260	NA	NA	41.1%/43.0%	NA	7
Heckman (2013) [19]	U.S.A.	Mixed	264/373	64/131/69	105/169/99	50.9%/49.2%	0.071	7
Lin (2011) [23]	Taiwan	Asian	627/1349	288/274/65	568/609/172	32.2%/35.3%	0.660	7
Xiong (2018) [26]	China	Asian	200/205	92/89/19	103/80/22	31.8%/30.2%	0.282	8
Yang (2018) [28]	China	Asian	550/548	236/237/77	244/251/53	35.5%/32.6%	0.317	8
Zhang (2012) [30]	China	Asian	1190/1637	487/552/151	652/769/216	35.9%/36.7%	0.649	8
**rs10757274**	GG/GA/AA							
Akinyemi (2018) [10]	Nigeria	African	82/247	NA	NA	14.8%/13.6%	NA	7
Hu (2009) [21]	China	Asian	353/430	101/193/59	154/202/74	44.1%/40.7%	0.579	7
Luke (2009) [24]	Austria	Caucasian	503/784	117/247/139	216/414/154	52.1%/46.0%	0.079	7
Xiong (2018) [26]	China	Asian	200/205	55/94/51	62/96/47	49.0%/46.3%	0.403	8
Yamagishi (2009) [27]	U.S.A.	African	218/3281	141/64/13	2033/1085/163	20.6%/21.5%	0.243	7
Yamagishi (2009) [27]	U.S.A.	Caucasian	306/9575	77/170/59	2551/4743/2281	47.1%/48.6%	0.405	7
Zee (2007) [29]	U.S.A.	Mixed	254/254	NA	NA	49.4%/49.2%	NA	7
Zhang (2012) [30]	China	Asian	1190/1664	341/556/293	492/842/330	48.0%/45.1%	0.376	8
**rs10757278**	GG/GA/AA							
Akinyemi (2018) [10]	Nigeria	African	82/247	NA	NA	11.0%/12.6%	NA	7
Bi (2015) [14]	China	Asian	116/118	38/49/29	56/47/15	46.1%/32.6%	0.307	8
Ding (2009) [16]	China	Asian	999/1055	378/431/190	384/497/174	40.6%/40.0%	0.538	8
Gschwendtner (2009) [17]	Germany	Caucasian	952/4262	NA	NA	50.5%/46.9%	NA	7
Heckman (2013) [19]	U.S.A.	Mixed	263/374	78/139/46	103/190/81	43.9%/47.1%	0.705	7
Helgadottir (2008) [20]	New Zealand	Caucasian	705/15012	NA	NA	46.8%/43.3%	NA	7
Lemmens (2009) [22]	Belgium	Caucasian	914/809	176/461/277	227/386/196	55.5%/48.1%	0.207	8
Olsson (2011) [25]	Sweden	Caucasian	834/665	222/415/197	191/342/132	48.5%/45.6%	0.343	8
Xiong (2018) [26]	China	Asian	200/205	53/95/52	59/99/47	49.8%/47.1%	0.656	8
Zhang (2012) [30]	China	Asian	986/1452	302/448/236	384/706/362	46.7%/49.2%	0.298	8

Abbreviation: NA, Not available.

### Overall and subgroup analyses

To investigate potential correlations between *ANRIL* variants and the risk of IS, six studies about rs1333040 polymorphism (2552 cases and 3359 controls), four studies about rs1333049 polymorphism (1543 cases and 2888 controls), five studies about rs2383206 polymorphism (2987 cases and 3600 controls), six studies about rs2383207 polymorphism (3793 cases and 8372 controls), seven studies about rs10757274 polymorphism (3106 cases and 16440 controls) and ten studies about rs10757278 polymorphism (6051 cases and 24199 controls) were enrolled for analyses. Significant associations with the risk of IS were detected for rs2383206 (recessive model: *P*=0.002, OR = 1.22, 95%CI 1.08–1.38, *I^2^* = 0%, FEM; allele model: *P*=0.003, OR = 0.90, 95%CI 0.84–0.96, *I^2^* = 0%, FEM) and rs10757274 (allele model: *P*=0.006, OR = 0.91, 95%CI 0.86–0.97, *I^2^* = 25%, FEM) variants in overall analyses. Further subgroup analyses by ethnicity of participants revealed that rs2383206, rs10757274 and rs10757278 variants were all significantly correlated with the risk of IS in Asians. Moreover, rs10757278 polymorphism was also significantly correlated with the risk of IS in Caucasians (see Table 2 and Supplementary Figure S1).

**Table 2:** Overall and subgroup analyses for *ANRIL* variants and IS

Polymorphisms	Population	Sample size	Dominant comparison		Recessive comparison		Additive comparison		Allele comparison	
			*P* value	OR (95%CI)	*P* value	OR (95%CI)	*P* value	OR (95%CI)	*P* value	OR (95%CI)
**rs1333040**	Overall	2552/3359	0.22	1.07 (0.96–1.20)	0.07	0.86 (0.73–1.01)	0.98	1.00 (0.90–1.12)	0.07	1.08 (0.99–1.16)
	Asian	1403/2098	0.50	1.05 (0.91–1.20)	0.21	0.86 (0.67–1.09)	0.95	1.00 (0.87–1.16)	0.28	1.06 (0.95–1.18)
**rs1333049**	Overall	1543/2888	0.10	0.89 (0.77–1.02)	0.20	1.11 (0.95–1.29)	0.68	1.03 (0.90–1.17)	0.07	0.92 (0.84–1.01)
	Asian	1392/2115	0.25	0.91 (0.79–1.06)	0.13	1.13 (0.96–1.34)	0.84	0.99 (0.86–1.13)	0.10	0.92 (0.84–1.02)
**rs2383206**	Overall	2987/3600	0.05	0.90 (0.80–1.00)	**0.002**	**1.22 (1.08–1.38)**	0.98	1.00 (0.83–1.20)	**0.003**	**0.90 (0.84–0.96)**
	Asian	2733/3346	0.05	0.90 (0.80–1.00)	**0.002**	**1.22 (1.08–1.38)**	0.98	1.00 (0.83–1.20)	**0.002**	**0.89 (0.83–0.96)**
**rs2383207**	Overall	3793/8372	0.64	1.02 (0.93–1.13)	0.79	0.98 (0.85–1.13)	0.79	0.99 (0.90–1.09)	0.21	1.04 (0.98–1.10)
	Asian	2567/3739	0.43	1.04 (0.94–1.16)	0.99	1.00 (0.76–1.32)	0.53	0.97 (0.87–1.07)	0.48	1.03 (0.95–1.11)
**rs10757274**	Overall	3106/16440	0.07	0.91 (0.82–1.01)	0.26	1.14 (0.91–1.44)	0.95	1.01 (0.84–1.20)	**0.006**	**0.91 (0.86–0.97)**
	Caucasian	809/10359	0.11	0.86 (0.71–1.03)	0.80	1.10 (0.54–2.21)	0.81	1.05 (0.71–1.53)	0.55	0.91 (0.67–1.23)
	Asian	1743/2299	0.11	0.89 (0.78–1.03)	**0.007**	**1.23 (1.06–1.44)**	0.80	1.04 (0.76–1.42)	**0.009**	**0.89 (0.81–0.97)**
**rs10757278**	Overall	6051/24199	0.35	0.90 (0.72–1.12)	0.10	1.16 (0.97–1.37)	0.20	0.95 (0.87–1.03)	0.05	0.91 (0.82–1.00)
	Caucasian	3405/20748	0.12	0.74 (0.51–1.08)	**0.001**	**1.31 (1.12–1.54)**	0.70	1.03 (0.89–1.18)	**<0.001**	**0.85 (0.80–0.90)**
	Asian	2301/2830	0.89	0.98 (0.77–1.26)	0.22	1.17 (0.91–1.50)	**0.03**	**0.88 (0.79–0.99)**	0.40	0.92 (0.76–1.11)

Abbreviation: NA, Not available.

The values in bold represent that there are statistically significant differences between cases and controls.

All investigated *ANRIL* variants contain a major allele (M) and a minor allele (m). In the current meta-analysis, dominant model is defined as MM versus Mm + mm, recessive model is defined as mm versus MM +Mm, Additive model is defined as Mm versus MM + mm, and the allele model is defined as M versus m.

### Sensitivity analyses

We performed sensitivity analyses to examine whether studies that deviated from HWE would impact the results of synthetic analyses. No alterations of results were detected in sensitivity analyses when we omitted one specific study each time, which suggested that our pooled results were statistically stable and reliable.

### Publication biases

Funnel plots were used to estimate publication biases. We did not find obvious asymmetry of funnel plots in any comparisons, which suggested that our findings were unlikely to be influenced by severe publication biases (see Supplementary Figure S2).

## Discussion

To the best of our knowledge, this is the most comprehensive meta-analysis on associations between *ANRIL* variants and the risk of IS. Our overall and subgroup analyses suggested that rs2383206, rs10757274 and rs10757278 variants were all significantly associated with an increased risk of IS in Asians. In addition, rs10757278 polymorphism was also significantly associated with an increased risk of IS in Caucasians. As shown in Supplementary Figure S1, for rs1333040, rs1333049, rs2383206 and rs2383207 variants, between-study heterogeneities were trivial, and thus pooled analyses were mainly performed with FEM. For rs10757274 and rs10757278 variants, however, obvious between-study heterogeneities were observed for recessive and additive comparisons, and thus REMs were employed for these analyses.

There are several points that need to be addressed about this meta-analysis. First, the exact function of ANRIL is still unclear, and therefore the underlying mechanisms of our positive findings need to be investigated by future investigations. Second, the pathogenic mechanism of IS is rather complex, and it is unlikely that a single genetic variant can significantly contribute to its development. So to better illustrate potential correlations of certain genetic variants with IS, we strongly recommend further studies to perform haplotype analyses and explore potential gene–gene interactions. Third, it is also worth noting that according to our findings, the associations between *ANRIL* variants and IS may be ethnic-specific, and this may explain why inconsistent results were observed in included original studies, especially when these studies were performed in different populations.

As with all meta-analysis, the present study certainly has some limitations. First, our results were derived from unadjusted analyses, and lack of further adjusted analyses for age, gender, smoking status and co-morbidity conditions (such as hypertension, diabetes, dyslipidemia, coronary artery disease and peripheral artery disease) may impact the reliability of our findings since the above-mentioned variables may also impact the individual susceptibility to IS [31,32]. Second, obvious heterogeneities were still found in several subgroup comparisons for rs10757274 and rs10757278 variants, which indicated that the controversial results of included studies could not be fully explained by differences in ethnic background, and other baseline characteristics of participants may also contribute to between-study heterogeneities [33,34]. Third, associations between *ANRIL* variants and IS may also be modified by gene–environment interactions. However, most eligible studies ignore these potential interactions, which impeded us to perform relevant analyses accordingly [35,36]. On account of above-mentioned limitations, our findings should be cautiously interpreted.

## Conclusions

In conclusion, our meta-analysis suggested that rs2383206, rs10757274 and rs10757278 variants may impact individual susceptibility to IS in Asians. Moreover, rs10757278 polymorphism may also impact individual susceptibility to IS in Caucasians. However, considering that the sample sizes of several comparisons were still relatively small, further well-designed studies with larger sample sizes are still warranted to confirm our findings.

## Supporting information

**Supplementary Figure S1:** 

**Supplementary Figure S2:** 

**Supplementary Materials:** 

## Abbreviations

ANRIL: antisense non-coding RNA in the INK4 locus
CI: confidence interval
FEM: fixed-effect model
HWE: Hardy–Weinberg equilibrium
IS: ischemic stroke
NOS: Newcastle–Ottawa scale
OR: odds ratio
REM: random-effect model
