# Supplementary material for: Effects of ANRIL variants on the risk of ischemic stroke: a meta-analysis
Source: Biosci Rep. 2019 May 15;39(5):BSR20182127. doi: 10.1042/BSR20182127 (PMC6522711; doi:10.1042/BSR20182127)
Supplement: Supplementary file 1 [file BSR-2018-2127_supp1.pdf]

PRISMA Checklist of Items to Include When Reporting A Systematic Review Involving a Network Meta-analysis

Section/Topic	Item #	Checklist Item	Reported on Page #
**TITLE**			
Title	1	Identify the report as a systematic review *incorporating a network meta-analysis (or related form of meta-analysis).*	1
**ABSTRACT**			
Structured summary	2	Provide a structured summary including, as applicable:<br>**Background:** main objectives<br>**Methods:** data sources; study eligibility criteria, participants, and interventions; study appraisal; and *synthesis methods, such as network meta-analysis.*<br>**Results:** number of studies and participants identified; summary estimates with corresponding confidence/credible intervals; *treatment rankings may also be discussed. Authors may choose to summarize pairwise comparisons against a chosen treatment included in their analyses for brevity.*<br>**Discussion/Conclusions:** limitations; conclusions and implications of findings.<br>**Other:** primary source of funding; systematic review registration number with registry name.	2
**INTRODUCTION**			
Rationale	3	Describe the rationale for the review in the context of what is already known, *including mention of why a network meta-analysis has been conducted.*	4
Objectives	4	Provide an explicit statement of questions being addressed, with reference to participants, interventions, comparisons, outcomes, and study design (PICOS).	4
**METHODS**			
Protocol and registration	5	Indicate whether a review protocol exists and if and where it can be accessed (e.g., Web address); and, if available, provide registration information, including registration number.	4
Eligibility criteria	6	Specify study characteristics (e.g., PICOS, length of follow-up) and report characteristics (e.g., years considered, language, publication status) used as criteria for eligibility, giving rationale. *Clearly describe eligible treatments included in the treatment network, and note whether any have been clustered or merged into the same node (with justification).*	5
Information sources	7	Describe all information sources (e.g., databases with dates of coverage, contact with study authors to identify additional studies) in the search and date last searched.	5
Search	8	Present full electronic search strategy for at least one database, including any limits used, such that it could be repeated.	5

Study selection	9	State the process for selecting studies (i.e., screening, eligibility, included in systematic review, and, if applicable, included in the meta-analysis).	6
Data collection process	10	Describe method of data extraction from reports (e.g., piloted forms, independently, in duplicate) and any processes for obtaining and confirming data from investigators.	5
Data items	11	List and define all variables for which data were sought (e.g., PICOS, funding sources) and any assumptions and simplifications made.	5
Risk of bias within individual studies	12	Describe methods used for assessing risk of bias of individual studies (including specification of whether this was done at the study or outcome level), and how this information is to be used in any data synthesis.	5
Summary measures	13	State the principal summary measures (e.g., risk ratio, difference in means). *Also describe the use of additional summary measures assessed, such as treatment rankings and surface under the cumulative ranking curve (SUCRA) values, as well as modified approaches used to present summary findings from meta-analyses.*	6
Planned methods of analysis	14	Describe the methods of handling data and combining results of studies for each network meta-analysis. This should include, but not be limited to:<br>• *Handling of multi-arm trials;*<br>• *Selection of variance structure;*<br>• *Selection of prior distributions in Bayesian analyses; and*<br>• *Assessment of model fit.*	6
Risk of bias across studies	15	Specify any assessment of risk of bias that may affect the cumulative evidence (e.g., publication bias, selective reporting within studies).	6
Additional analyses	16	Describe methods of additional analyses if done, indicating which were pre-specified. This may include, but not be limited to, the following:<br>• Sensitivity or subgroup analyses;<br>• Meta-regression analyses;<br>• *Alternative formulations of the treatment network; and*<br>• *Use of alternative prior distributions for Bayesian analyses (if applicable).*	6
**RESULTS†**			
Study selection	17	Give numbers of studies screened, assessed for eligibility, and included in the review, with reasons for exclusions at each stage, ideally with a flow diagram.	6
Study characteristics	18	For each study, present characteristics for which data were extracted (e.g., study size, PICOS, follow-up period) and provide the citations.	6

Risk of bias within studies	19	Present data on risk of bias of each study and, if available, any outcome level assessment.	7
Results of individual studies	20	For all outcomes considered (benefits or harms), present, for each study: 1) simple summary data for each intervention group, and 2) effect estimates and confidence intervals. *Modified approaches may be needed to deal with information from larger networks.*	7
Synthesis of results	21	Present results of each meta-analysis done, including confidence/credible intervals. *In larger networks, authors may focus on comparisons versus a particular comparator (e.g. placebo or standard care), with full findings presented in an appendix. League tables and forest plots may be considered to summarize pairwise comparisons.* If additional summary measures were explored (such as treatment rankings), these should also be presented.	7
Risk of bias across studies	22	Present results of any assessment of risk of bias across studies for the evidence base being studied.	7
Results of additional analyses	23	Give results of additional analyses, if done (e.g., sensitivity or subgroup analyses, meta-regression analyses, *alternative network geometries studied, alternative choice of prior distributions for Bayesian analyses,* and so forth).	7
**DISCUSSION**			
Summary of evidence	24	Summarize the main findings, including the strength of evidence for each main outcome; consider their relevance to key groups (e.g., healthcare providers, users, and policy-makers).	8
Limitations	25	Discuss limitations at study and outcome level (e.g., risk of bias), and at review level (e.g., incomplete retrieval of identified research, reporting bias). *Comment on the validity of the assumptions, such as transitivity and consistency. Comment on any concerns regarding network geometry (e.g., avoidance of certain comparisons).*	9
Conclusions	26	Provide a general interpretation of the results in the context of other evidence, and implications for future research.	10
**FUNDING**			
Funding	27	Describe sources of funding for the systematic review and other support (e.g., supply of data); role of funders for the systematic review. This should also include information regarding whether funding has been received from manufacturers of treatments in the network and/or whether some of the authors are content experts with professional conflicts of interest that could affect use of treatments in the network.	10

PICOS = population, intervention, comparators, outcomes, study design.
* Text in italics indicateS wording specific to reporting of network meta-analyses that has been added to guidance from the PRISMA statement.
† Authors may wish to plan for use of appendices to present all relevant information in full detail for items in this section.

Forest plots of investigated polymorphisms

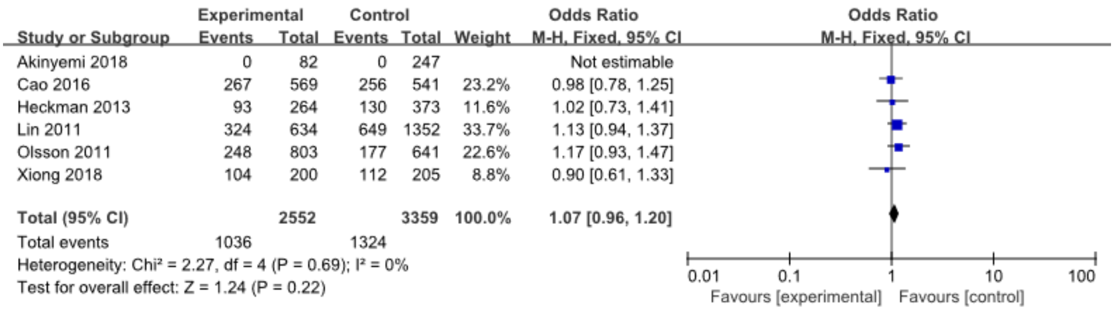

Forest plot of the **rs1333040** polymorphism and IS under dominant comparison.

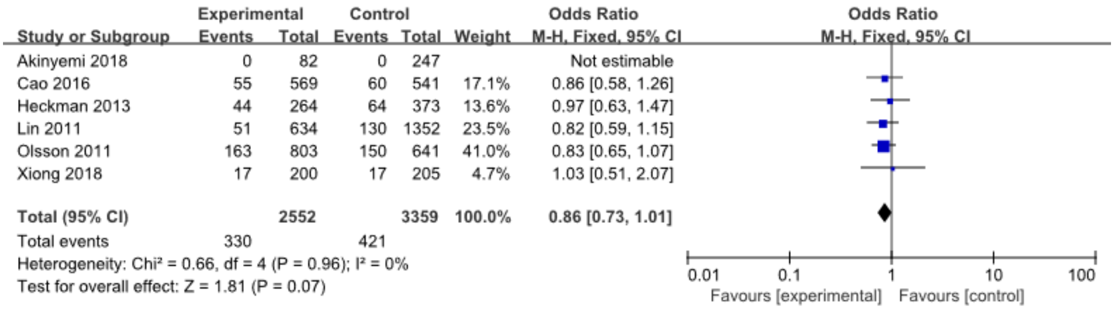

Forest plot of the **rs1333040** polymorphism and IS under recessive comparison.

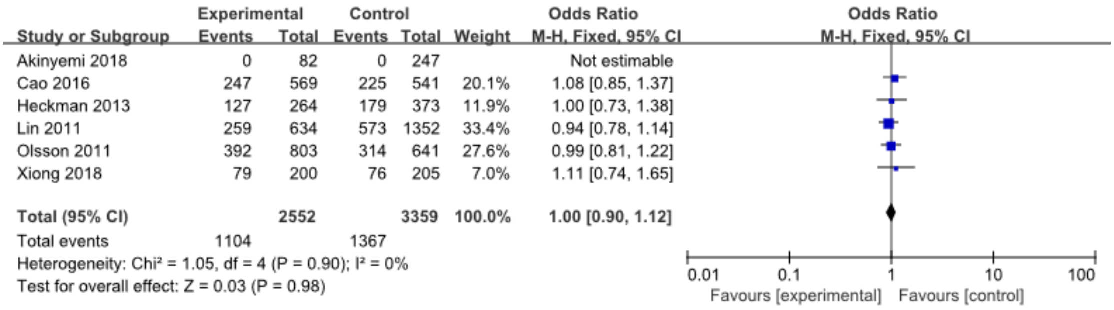

Forest plot of the **rs1333040** polymorphism and IS under additive comparison.

Study or Subgroup	Experimental Events	Experimental Total	Control Events	Control Total	Weight	Odds Ratio M-H, Fixed, 95% CI
Akinyemi 2018	95	164	282	494	4.9%	1.04 [0.72, 1.48]
Cao 2016	781	1138	737	1082	19.7%	1.02 [0.86, 1.22]
Heckman 2013	313	528	439	746	12.3%	1.02 [0.81, 1.28]
Lin 2011	907	1268	1871	2704	28.3%	1.12 [0.97, 1.30]
Olsson 2011	888	1606	668	1282	27.7%	1.14 [0.98, 1.32]
Xiong 2018	287	400	300	410	7.0%	0.93 [0.68, 1.27]
**Total (95% CI)**		**5104**		**6718**	**100.0%**	**1.08 [0.99, 1.16]**
Total events	3271		4297			

Heterogeneity: Chi² = 2.21, df = 5 (P = 0.82); I² = 0%
Test for overall effect: Z = 1.81 (P = 0.07)

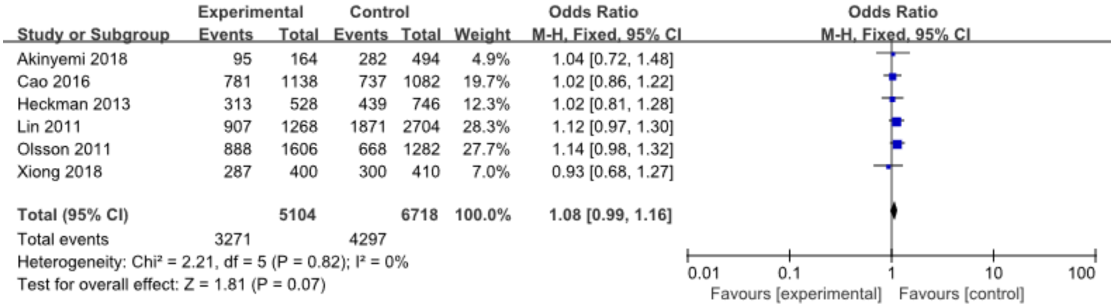

Forest plot of the **rs1333040** polymorphism and IS under allele comparison.

Study or Subgroup	Experimental Events	Experimental Total	Control Events	Control Total	Weight	Odds Ratio M-H, Fixed, 95% CI
Haslacher 2016	33	151	218	773	13.8%	0.71 [0.47, 1.08]
Lin 2011	176	642	395	1361	45.4%	0.92 [0.75, 1.14]
Xiong 2018	53	200	56	205	10.1%	0.96 [0.62, 1.49]
Yang 2018	162	550	176	549	30.7%	0.88 [0.68, 1.14]
**Total (95% CI)**		**1543**		**2888**	**100.0%**	**0.89 [0.77, 1.02]**
Total events	424		845			

Heterogeneity: Chi² = 1.34, df = 3 (P = 0.72); I² = 0%
Test for overall effect: Z = 1.66 (P = 0.10)

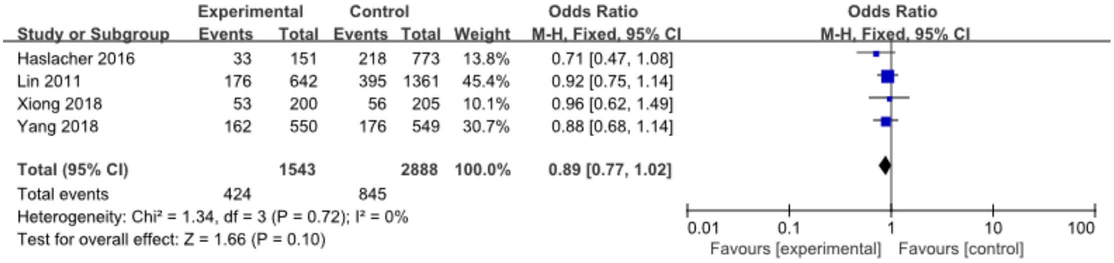

Forest plot of the **rs1333049** polymorphism and IS under dominant comparison.

Study or Subgroup	Experimental Events	Experimental Total	Control Events	Control Total	Weight	Odds Ratio M-H, Fixed, 95% CI
Haslacher 2016	34	151	183	773	14.9%	0.94 [0.62, 1.42]
Lin 2011	153	642	311	1361	48.9%	1.06 [0.85, 1.32]
Xiong 2018	51	200	47	205	11.1%	1.15 [0.73, 1.81]
Yang 2018	122	550	100	549	25.1%	1.28 [0.95, 1.72]
**Total (95% CI)**		**1543**		**2888**	**100.0%**	**1.11 [0.95, 1.29]**
Total events	360		641			

Heterogeneity: Chi² = 1.74, df = 3 (P = 0.63); I² = 0%
Test for overall effect: Z = 1.28 (P = 0.20)

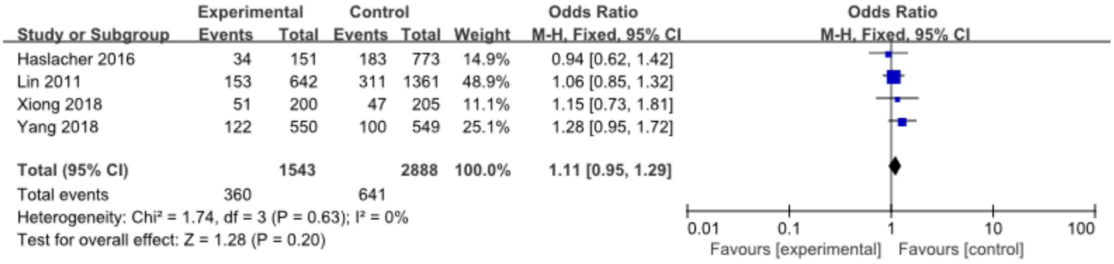

Forest plot of the **rs1333049** polymorphism and IS under recessive comparison.

Study or Subgroup	Experimental Events	Experimental Total	Control Events	Control Total	Weight	Odds Ratio M-H, Fixed, 95% CI
Haslacher 2016	84	151	374	773	11.7%	1.34 [0.94, 1.90]
Lin 2011	313	642	655	1361	46.5%	1.03 [0.85, 1.24]
Xiong 2018	96	200	102	205	11.3%	0.93 [0.63, 1.38]
Yang 2018	266	550	273	549	30.5%	0.95 [0.75, 1.20]
**Total (95% CI)**		**1543**		**2888**	**100.0%**	**1.03 [0.90, 1.17]**
Total events	759		1404			

Heterogeneity: Chi² = 2.87, df = 3 (P = 0.41); I² = 0%
Test for overall effect: Z = 0.42 (P = 0.68)

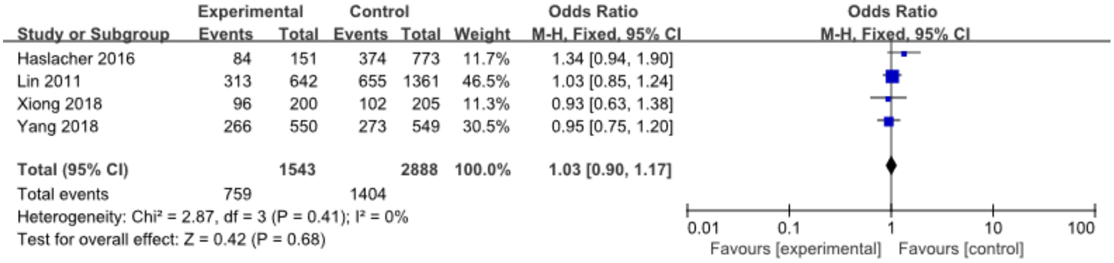

Forest plot of the **rs1333049** polymorphism and IS under additive comparison.

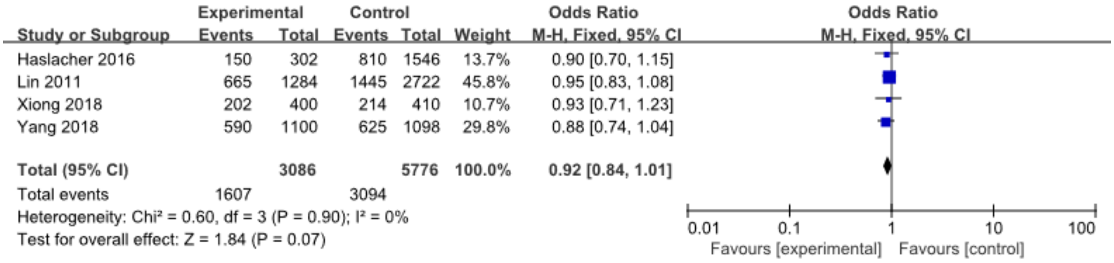

Forest plot of the **rs1333049** polymorphism and IS under allele comparison.

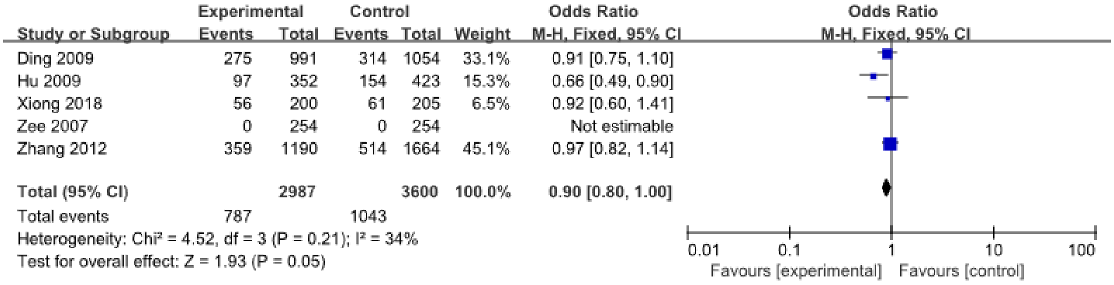

Forest plot of the **rs2383206** polymorphism and IS under dominant comparison.

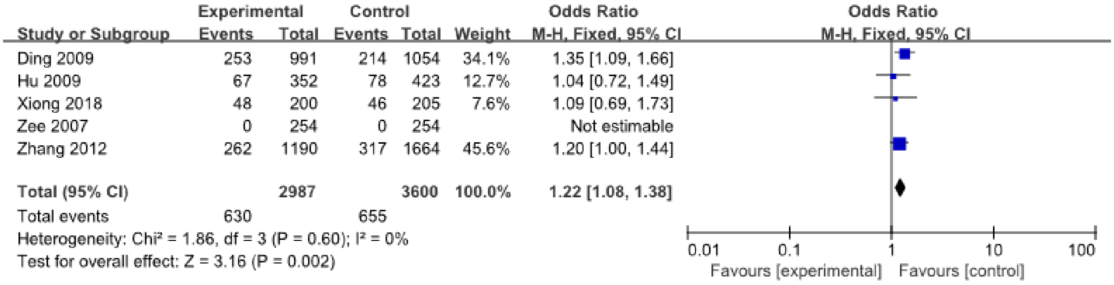

Forest plot of the **rs2383206** polymorphism and IS under recessive comparison.

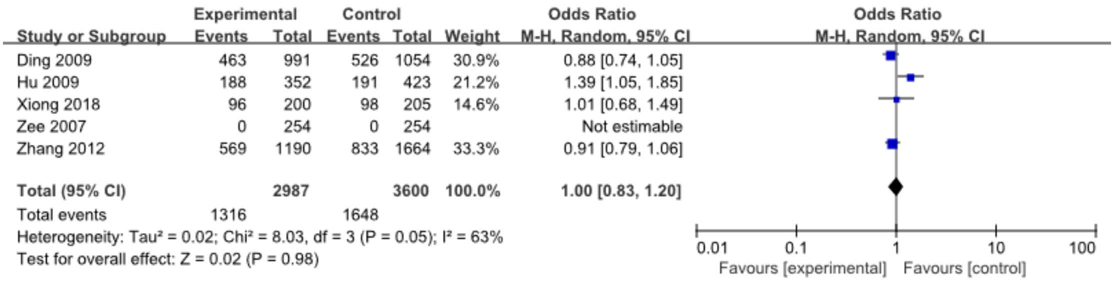

Forest plot of the **rs2383206** polymorphism and IS under additive comparison.

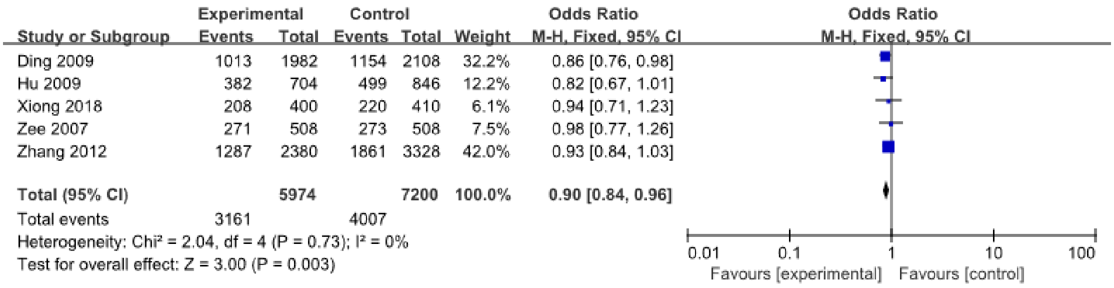

Forest plot of the **rs2383206** polymorphism and IS under allele comparison.

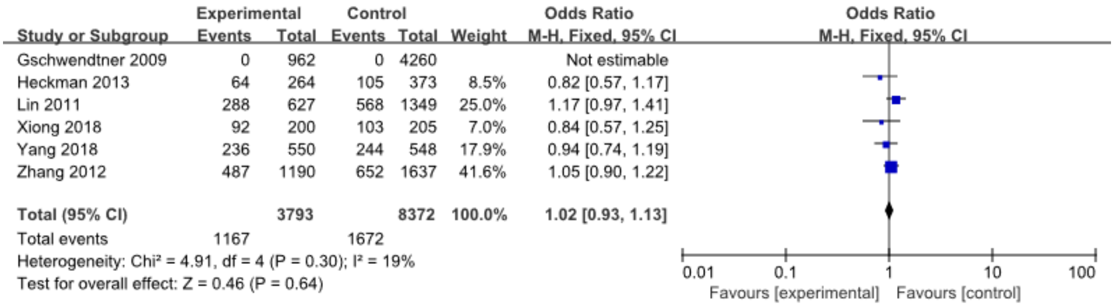

Forest plot of the **rs2383207** polymorphism and IS under dominant comparison.

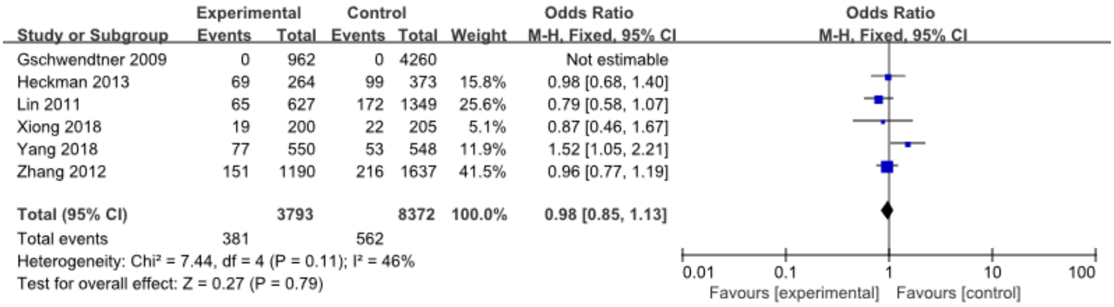

Forest plot of the **rs2383207** polymorphism and IS under recessive comparison.

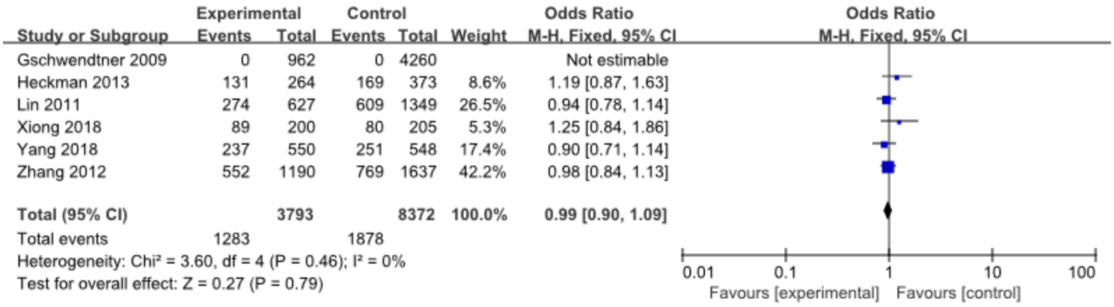

Forest plot of the **rs2383207** polymorphism and IS under additive comparison.

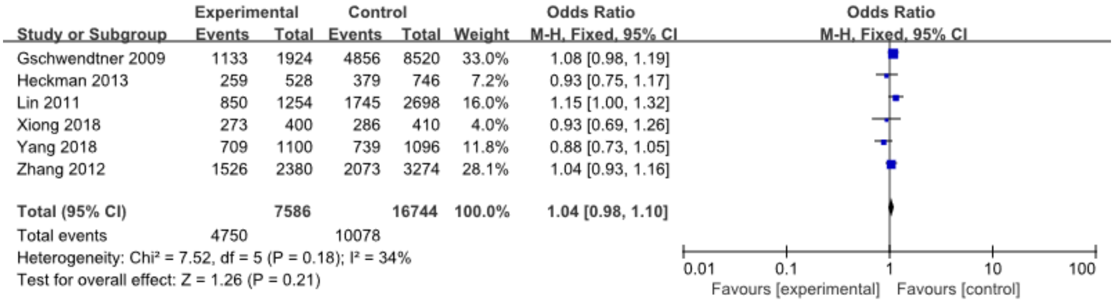

Forest plot of the **rs2383207** polymorphism and IS under allele comparison.

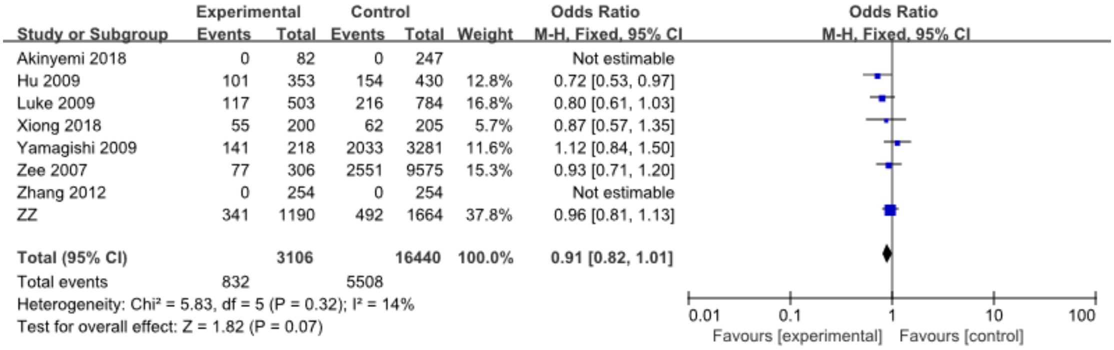

Forest plot of the **rs10757274** polymorphism and IS under dominant comparison.

Study or Subgroup	Experimental Events	Experimental Total	Control Events	Control Total	Weight	Odds Ratio M-H, Random, 95% CI
Akinyemi 2018	0	82	0	247		Not estimable
Hu 2009	59	353	74	430	15.6%	0.97 [0.66, 1.41]
Luke 2009	139	503	154	784	19.7%	1.56 [1.20, 2.03]
Xiong 2018	51	200	47	205	13.1%	1.15 [0.73, 1.81]
Yamagishi 2009	13	218	163	3281	9.9%	1.21 [0.68, 2.17]
Zee 2007	59	306	2281	9575	18.8%	0.76 [0.57, 1.02]
Zhang 2012	0	254	0	254		Not estimable
ZZ	293	1190	330	1664	22.9%	1.32 [1.10, 1.58]
**Total (95% CI)**		**3106**		**16440**	**100.0%**	**1.14 [0.91, 1.44]**
Total events	614		3049			

Heterogeneity: Tau² = 0.05; Chi² = 15.75, df = 5 (P = 0.008); I² = 68%
Test for overall effect: Z = 1.13 (P = 0.26)

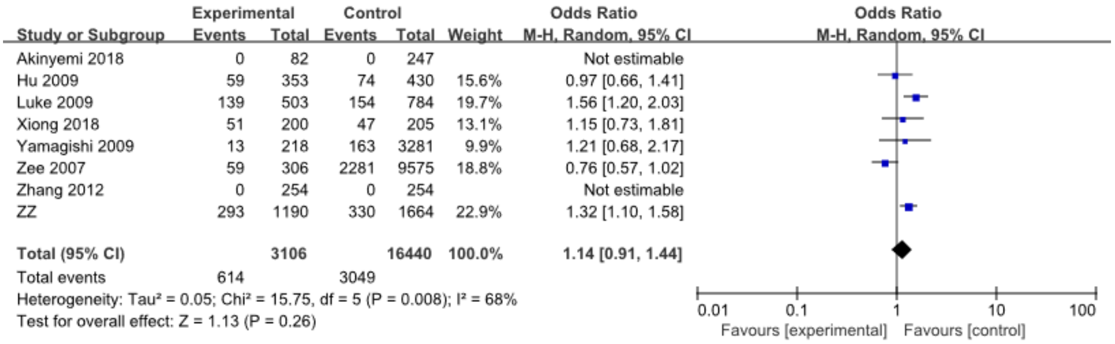

Forest plot of the **rs10757274** polymorphism and IS under recessive comparison.

Study or Subgroup	Experimental Events	Experimental Total	Control Events	Control Total	Weight	Odds Ratio M-H, Random, 95% CI
Akinyemi 2018	0	82	0	247		Not estimable
Hu 2009	193	353	202	430	15.6%	1.36 [1.03, 1.81]
Luke 2009	247	503	414	784	18.3%	0.86 [0.69, 1.08]
Xiong 2018	94	200	96	205	11.5%	1.01 [0.68, 1.49]
Yamagishi 2009	64	218	1085	3281	14.8%	0.84 [0.62, 1.14]
Zee 2007	170	306	4743	9575	18.0%	1.27 [1.01, 1.60]
Zhang 2012	0	254	0	254		Not estimable
ZZ	556	1190	842	1664	21.8%	0.86 [0.74, 0.99]
**Total (95% CI)**		**3106**		**16440**	**100.0%**	**1.01 [0.84, 1.20]**
Total events	1324		7382			

Heterogeneity: Tau² = 0.03; Chi² = 15.62, df = 5 (P = 0.008); I² = 68%
Test for overall effect: Z = 0.07 (P = 0.95)

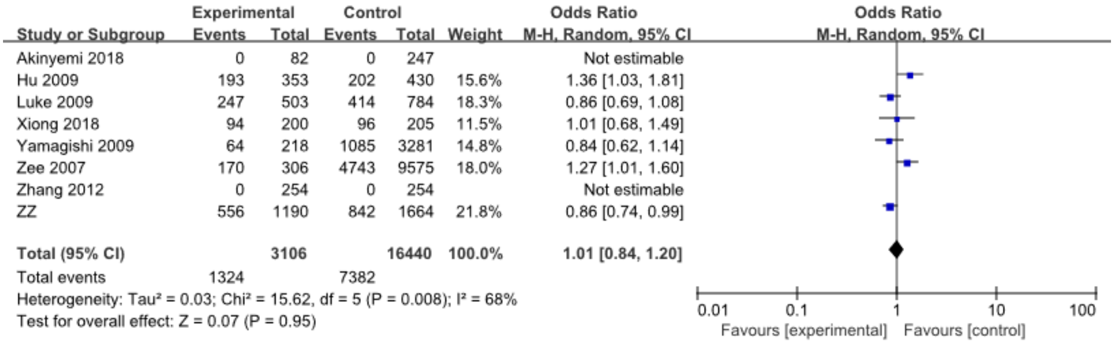

Forest plot of the **rs10757274** polymorphism and IS under additive comparison.

Study or Subgroup	Experimental Events	Experimental Total	Control Events	Control Total	Weight	Odds Ratio M-H, Fixed, 95% CI
Akinyemi 2018	140	164	427	494	1.6%	0.92 [0.55, 1.51]
Hu 2009	395	706	510	860	10.3%	0.87 [0.71, 1.07]
Luke 2009	481	1006	846	1568	17.6%	0.78 [0.67, 0.92]
Xiong 2018	204	400	220	410	5.4%	0.90 [0.68, 1.18]
Yamagishi 2009	346	436	5151	6562	6.7%	1.05 [0.83, 1.34]
Zee 2007	324	612	9845	19150	14.6%	1.06 [0.90, 1.25]
Zhang 2012	257	508	258	508	6.5%	0.99 [0.78, 1.27]
ZZ	1238	2380	1826	3328	37.2%	0.89 [0.80, 0.99]
**Total (95% CI)**		**6212**		**32880**	**100.0%**	**0.91 [0.86, 0.97]**
Total events	3385		19083			

Heterogeneity: Chi² = 9.31, df = 7 (P = 0.23); I² = 25%
Test for overall effect: Z = 2.77 (P = 0.006)

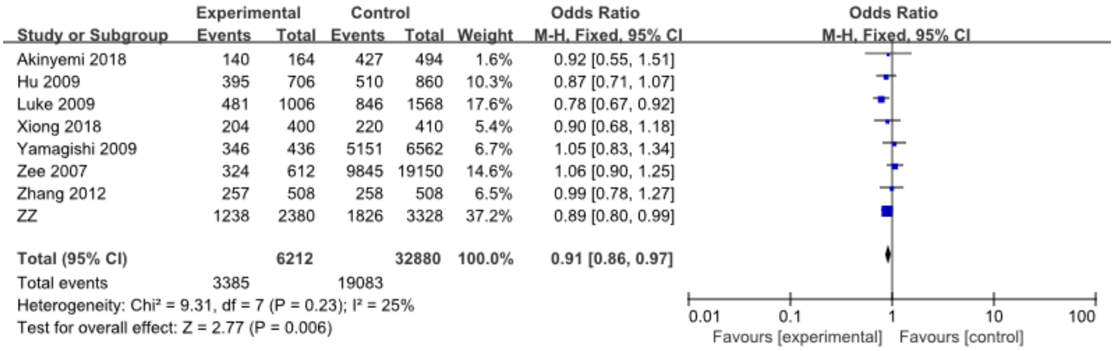

Forest plot of the **rs10757274** polymorphism and IS under allele comparison.

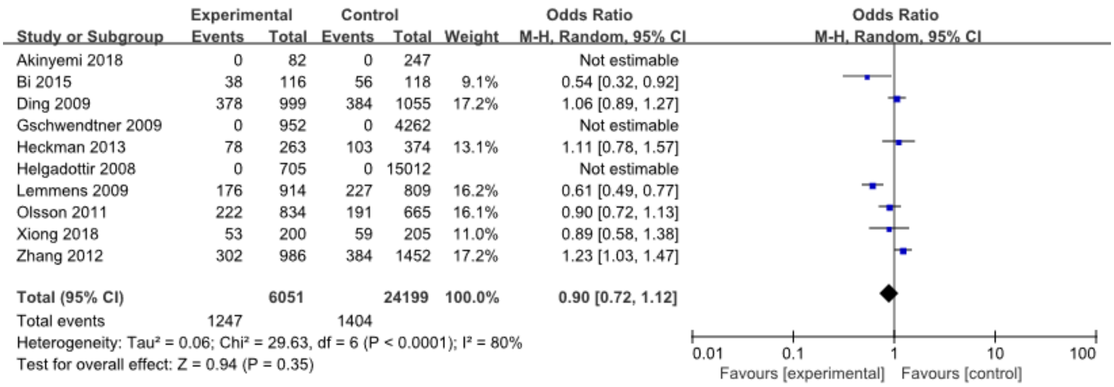

Forest plot of the **rs10757278** polymorphism and IS under dominant comparison.

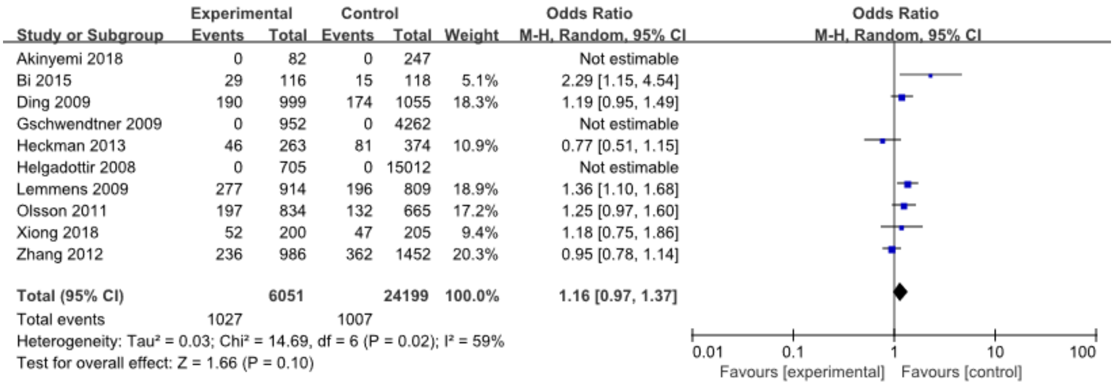

Forest plot of the **rs10757278** polymorphism and IS under recessive comparison.

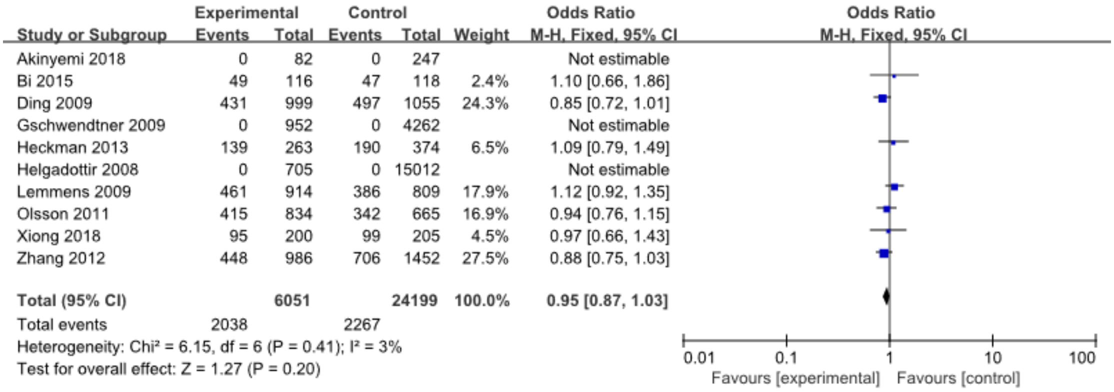

Forest plot of the **rs10757278** polymorphism and IS under additive comparison.

Study or Subgroup	Experimental Events	Experimental Total	Control Events	Control Total	Weight	Odds Ratio M-H, Random, 95% CI
Akinyemi 2018	146	164	432	494	2.6%	1.16 [0.67, 2.03]
Bi 2015	125	232	159	236	4.8%	0.57 [0.39, 0.82]
Ding 2009	1187	1998	1265	2110	12.6%	0.98 [0.86, 1.11]
Gschwendtner 2009	942	1904	4526	8524	13.7%	0.86 [0.78, 0.96]
Heckman 2013	295	526	396	748	8.7%	1.14 [0.91, 1.42]
Helgadottir 2008	750	1410	16934	30024	13.4%	0.88 [0.79, 0.98]
Lemmens 2009	813	1828	840	1618	12.3%	0.74 [0.65, 0.85]
Olsson 2011	859	1668	724	1330	11.8%	0.89 [0.77, 1.03]
Xiong 2018	201	400	217	410	7.1%	0.90 [0.68, 1.18]
Zhang 2012	1052	1972	1474	2904	13.1%	1.11 [0.99, 1.24]
**Total (95% CI)**		**12102**		**48398**	**100.0%**	**0.91 [0.82, 1.00]**
Total events	6370		26967			

Heterogeneity: Tau² = 0.02; Chi² = 33.84, df = 9 (P < 0.0001); I² = 73%

Test for overall effect: Z = 1.93 (P = 0.05)

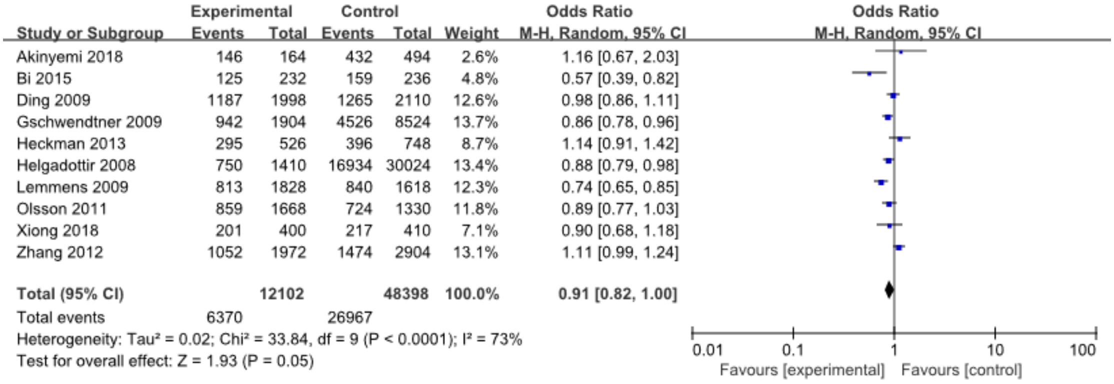

Forest plot of the **rs10757278** polymorphism and IS under allele comparison.

Funnel plots of investigated polymorphisms

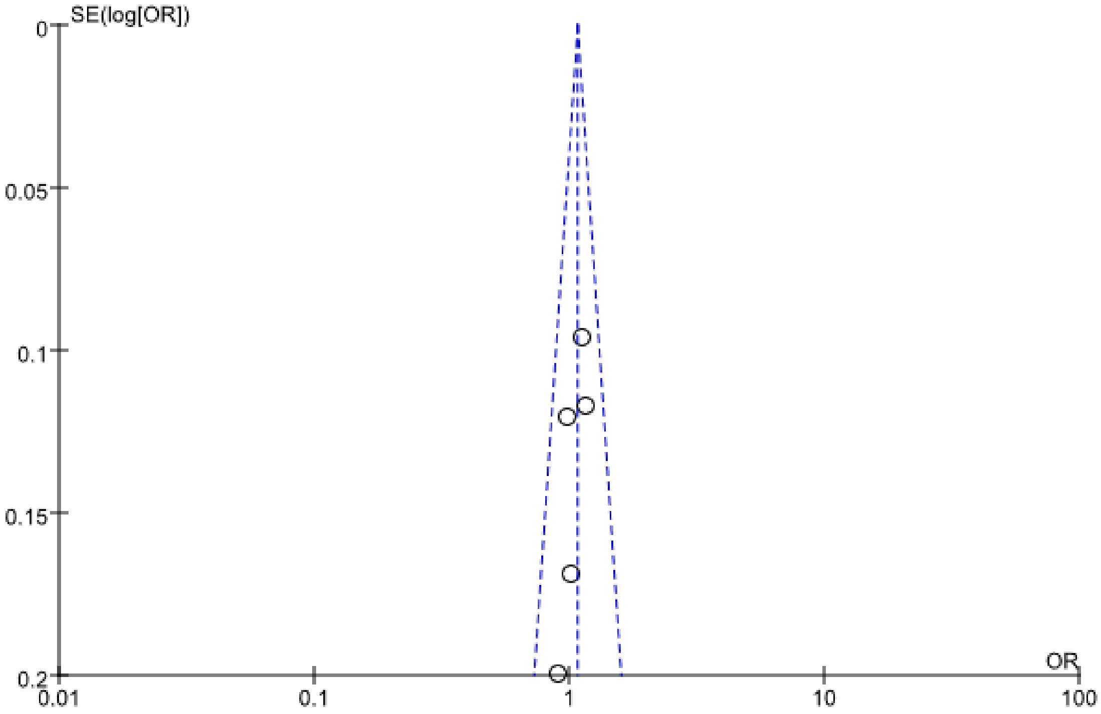

Funnel plot of the **rs1333040** polymorphism and IS under dominant comparison.

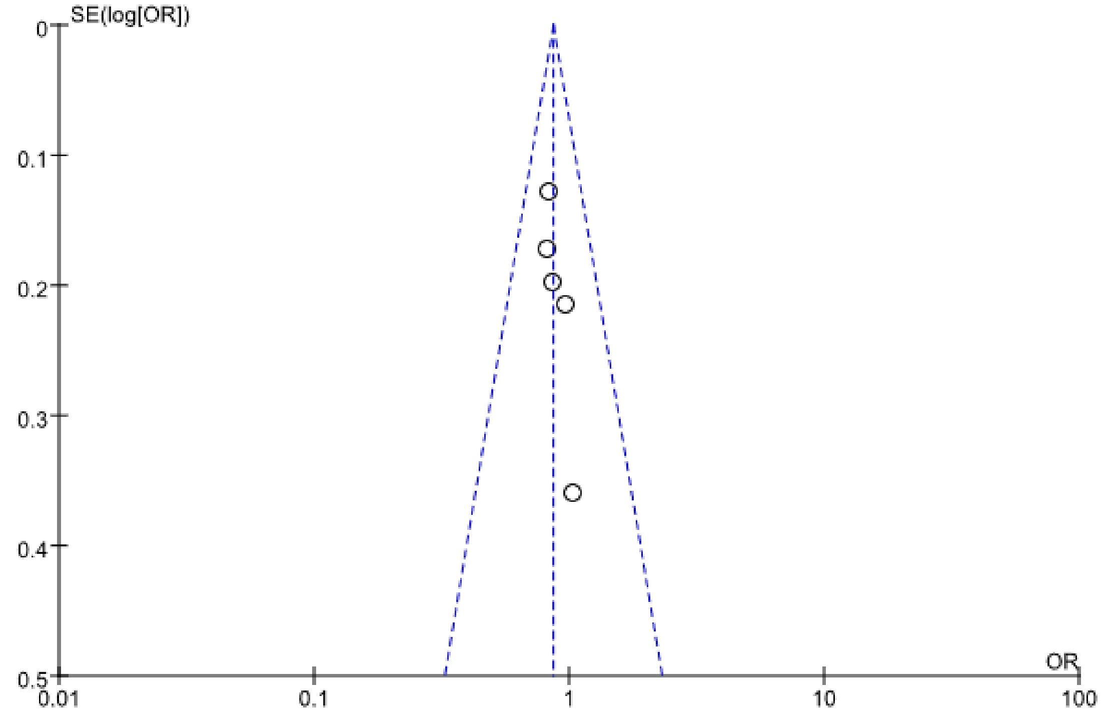

Funnel plot of the **rs1333040** polymorphism and IS under recessive comparison.

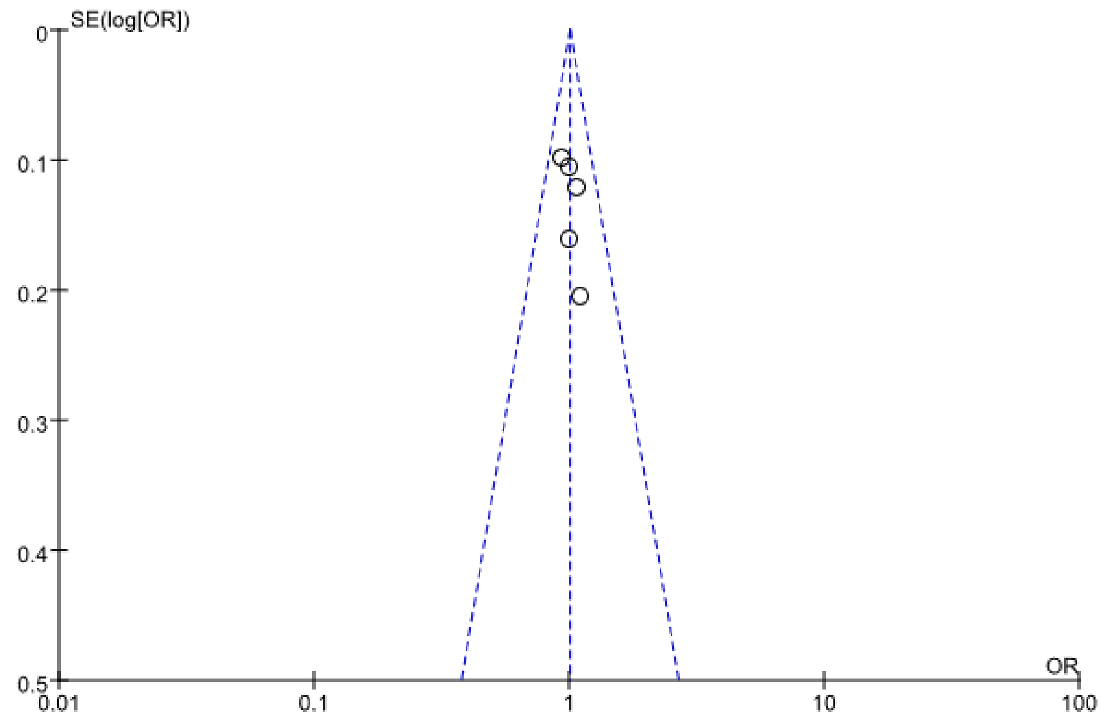

Funnel plot of the **rs1333040** polymorphism and IS under additive comparison.

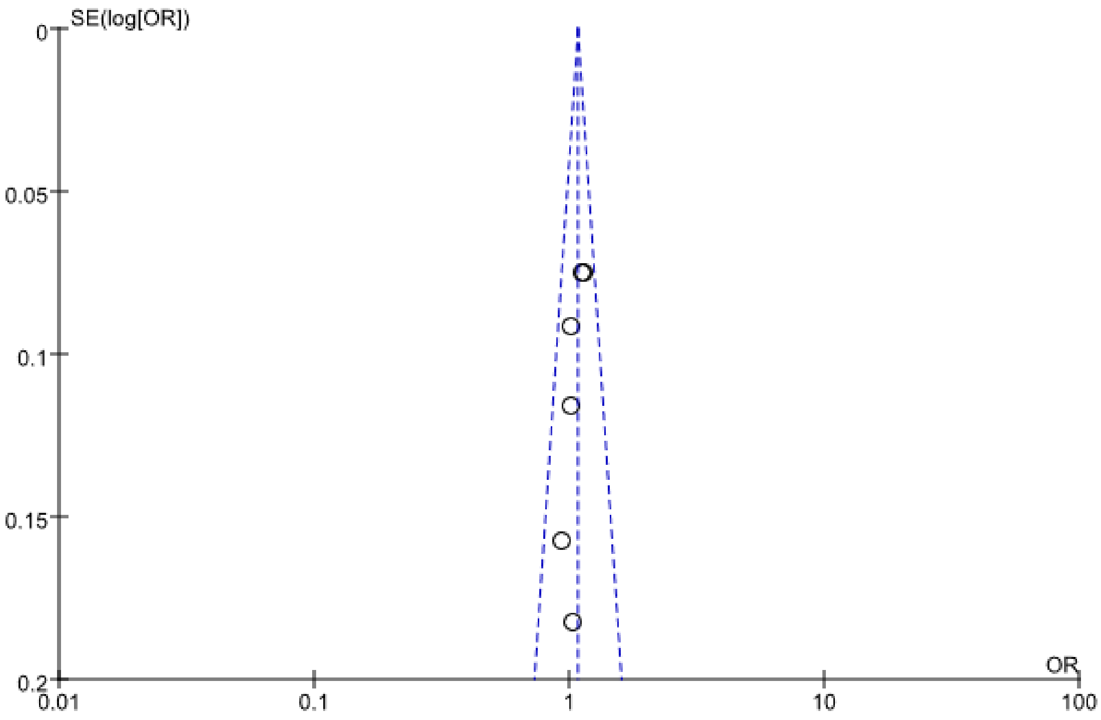

Funnel plot of the **rs1333040** polymorphism and IS under allele comparison.

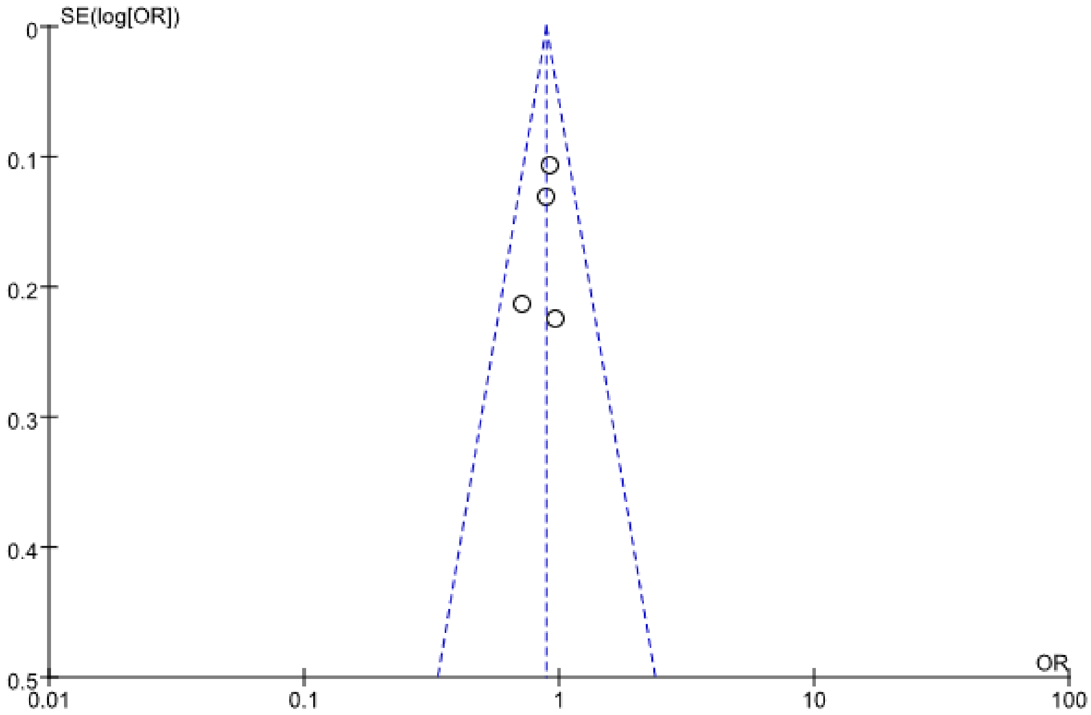

Funnel plot of the **rs1333049** polymorphism and IS under dominant comparison.

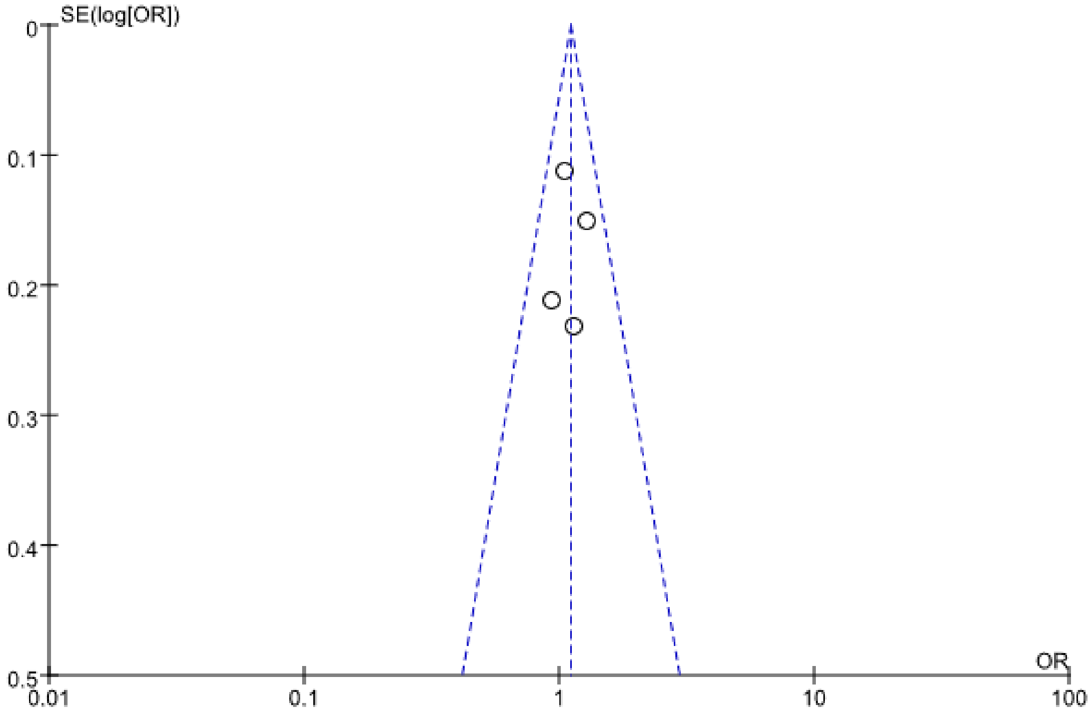

Funnel plot of the **rs1333049** polymorphism and IS under recessive comparison.

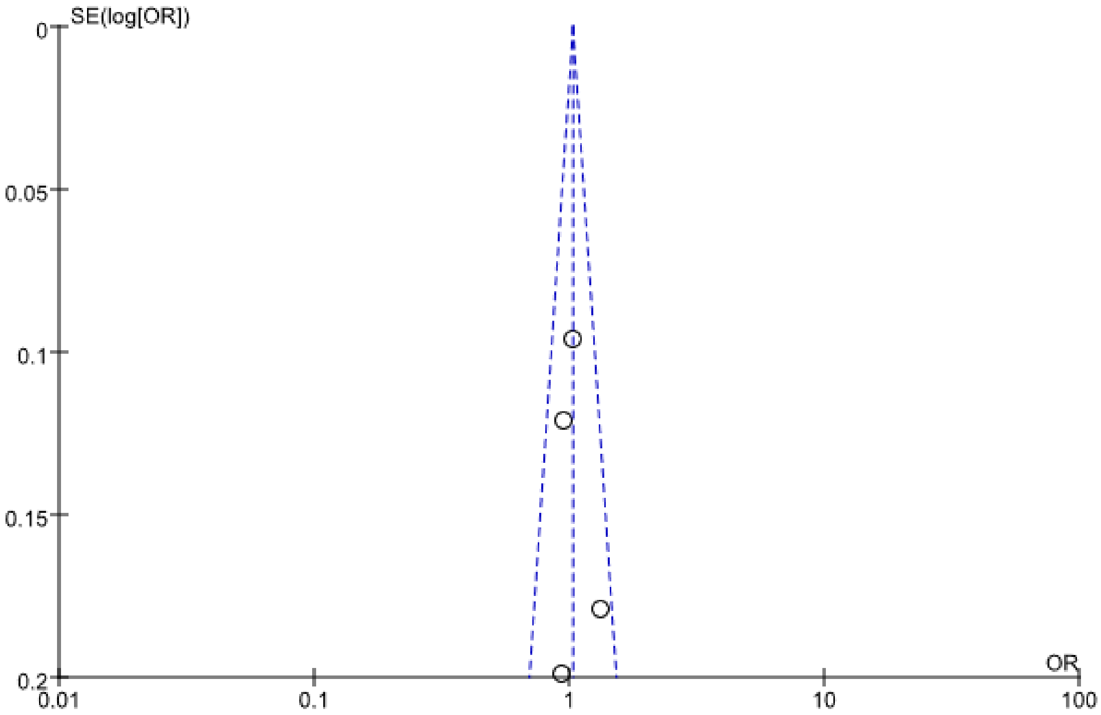

Funnel plot of the **rs1333049** polymorphism and IS under additive comparison.

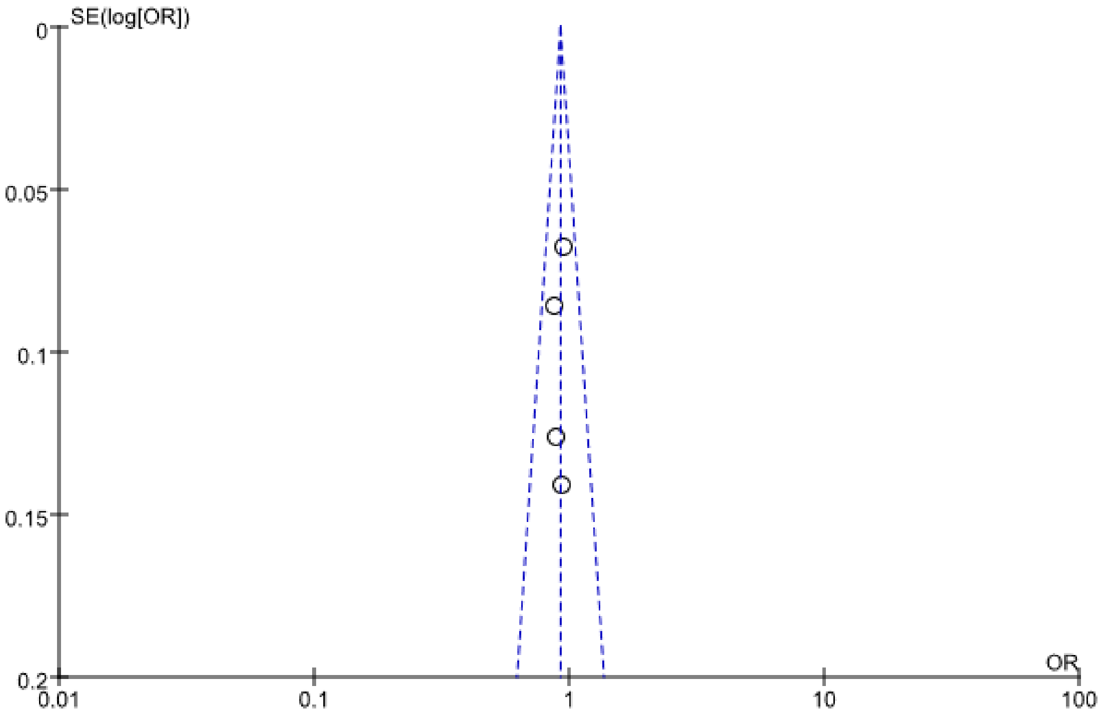

Funnel plot of the **rs1333049** polymorphism and IS under allele comparison.

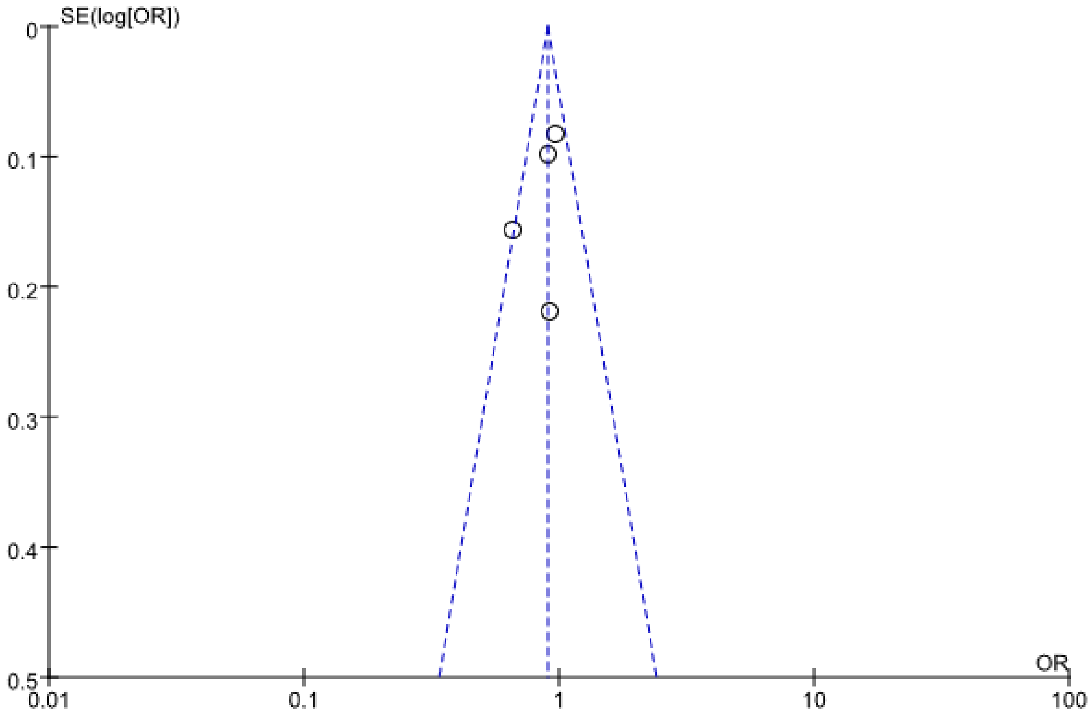

Funnel plot of the **rs2383206** polymorphism and IS under dominant comparison.

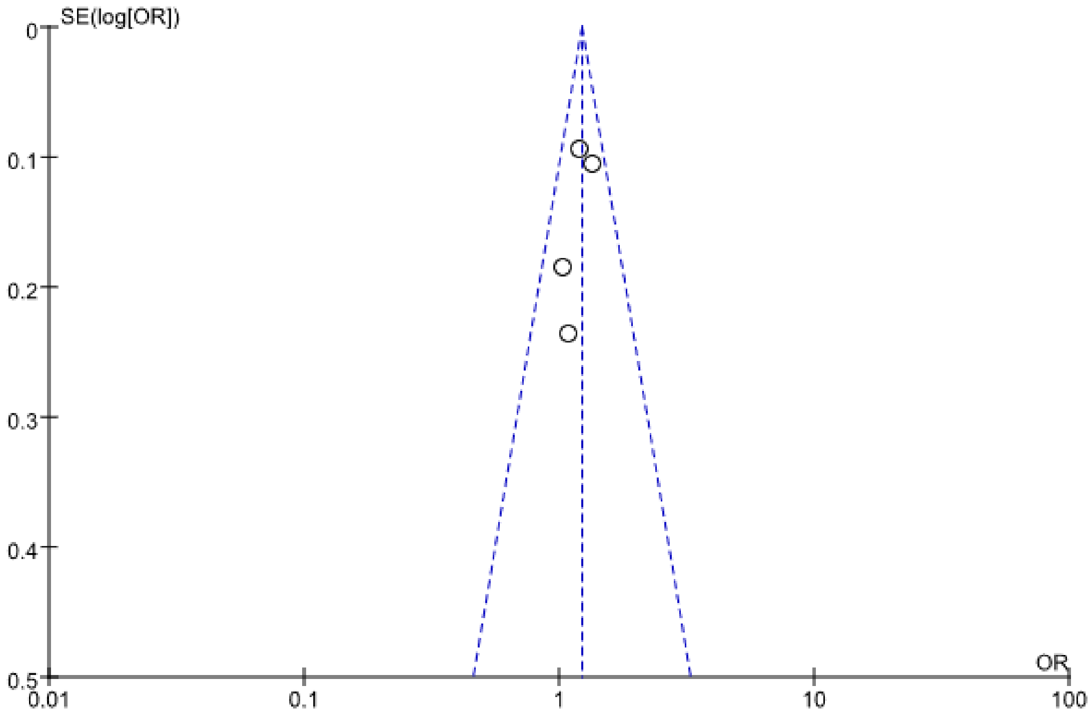

Funnel plot of the **rs2383206** polymorphism and IS under recessive comparison.

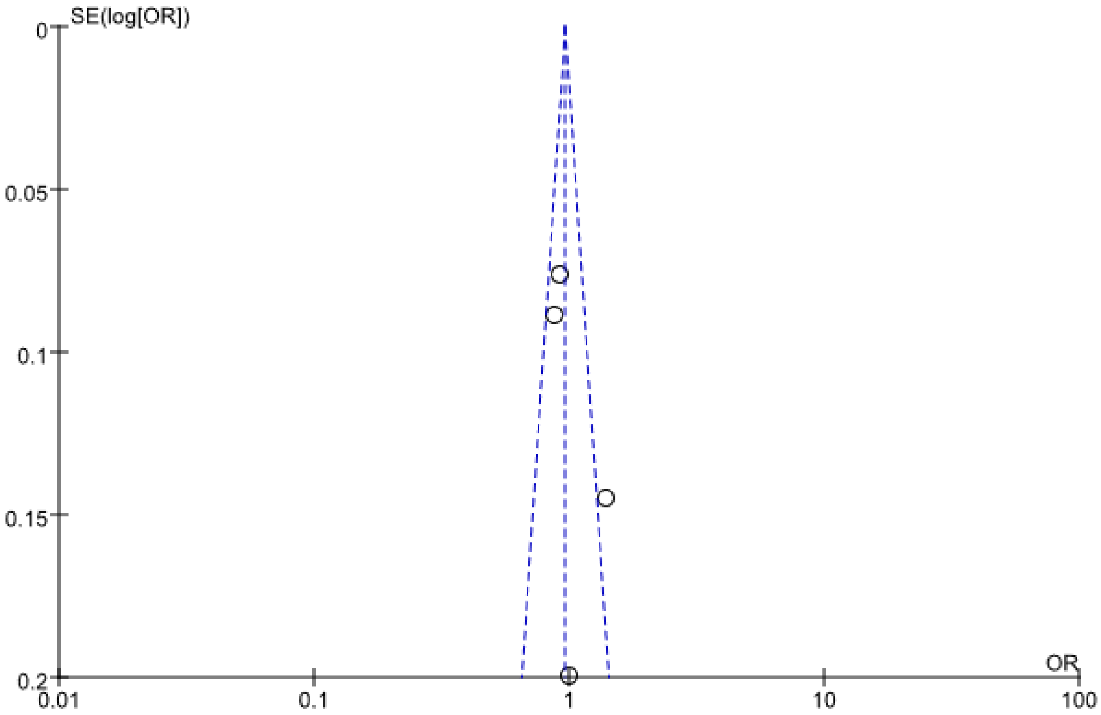

Funnel plot of the **rs2383206** polymorphism and IS under additive comparison.

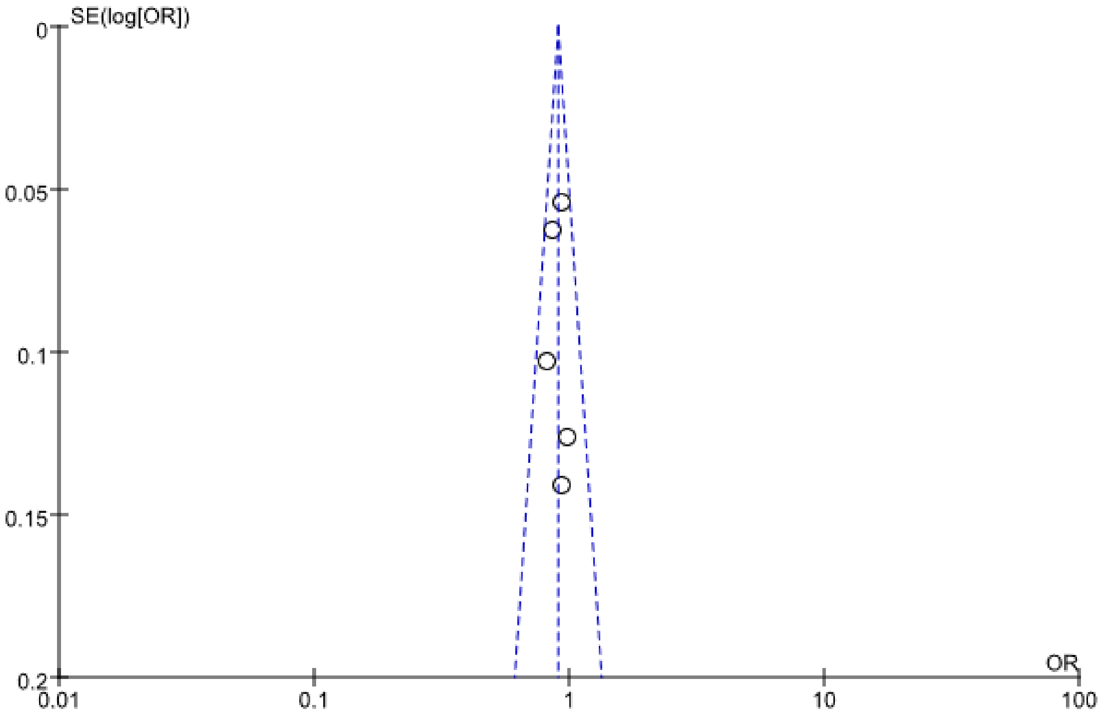

Funnel plot of the **rs2383206** polymorphism and IS under allele comparison.

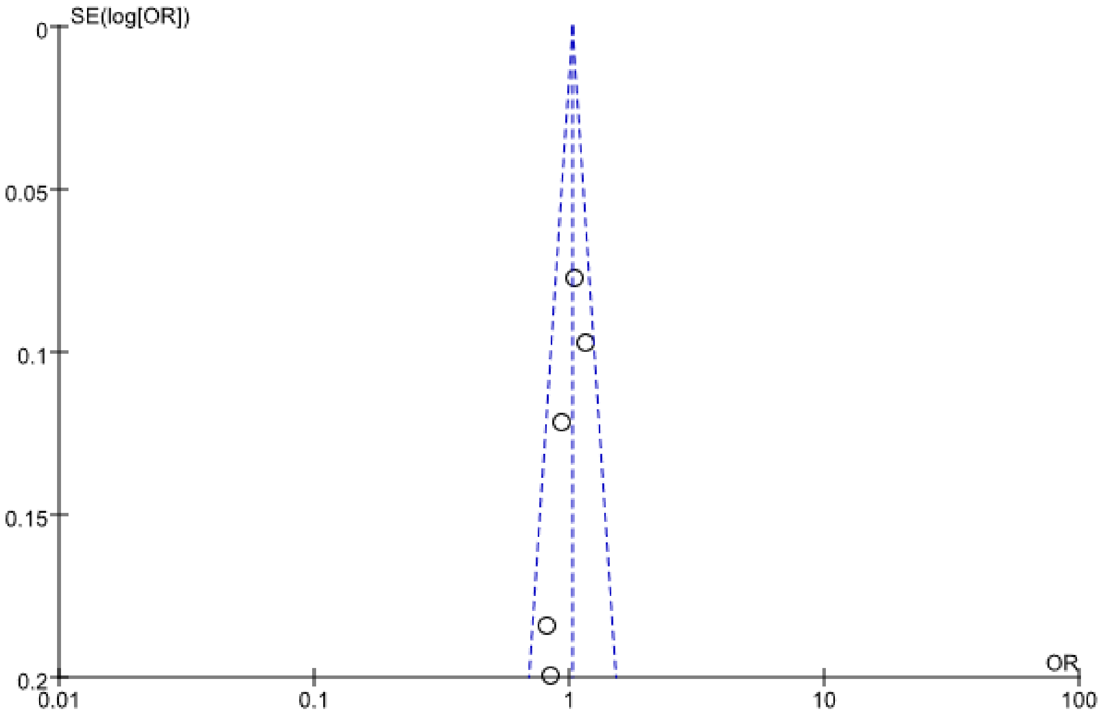

Funnel plot of the **rs2383207** polymorphism and IS under dominant comparison.

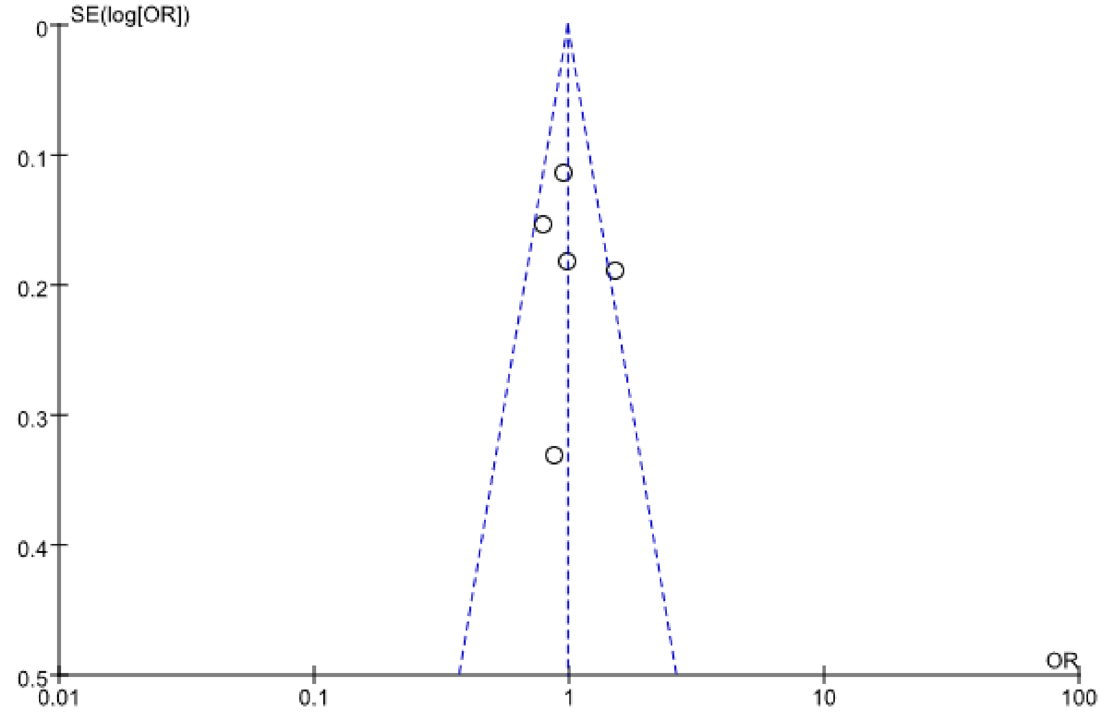

Funnel plot of the **rs2383207** polymorphism and IS under recessive comparison.

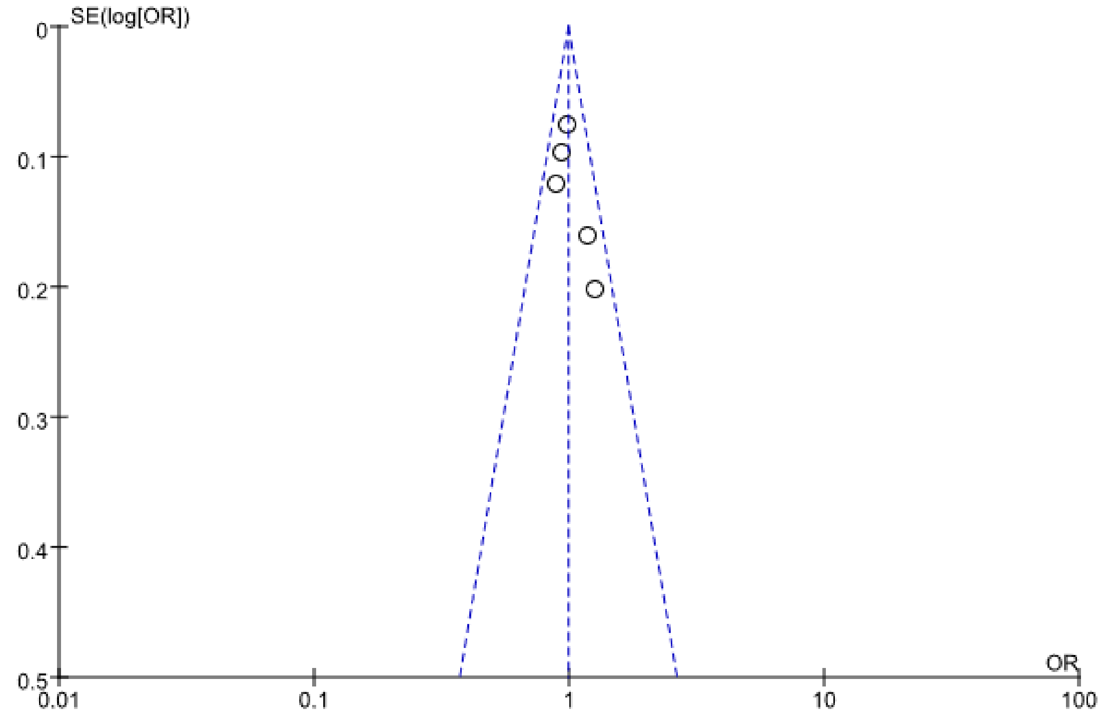

Funnel plot of the **rs2383207** polymorphism and IS under additive comparison.

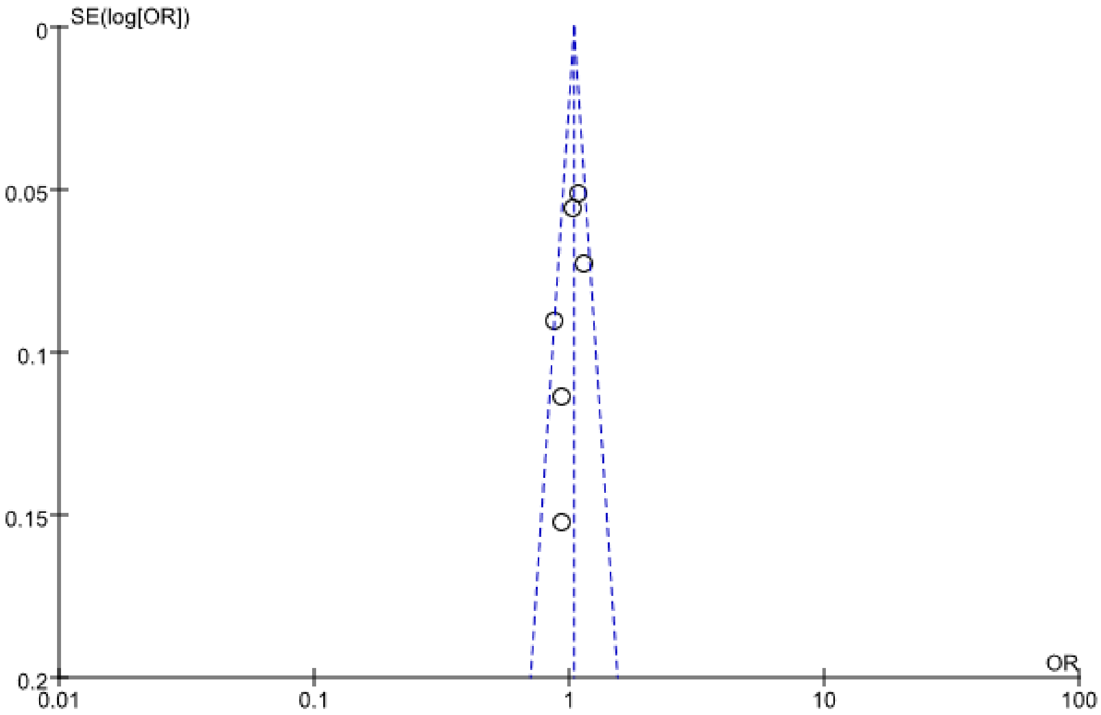

Funnel plot of the **rs2383207** polymorphism and IS under allele comparison.

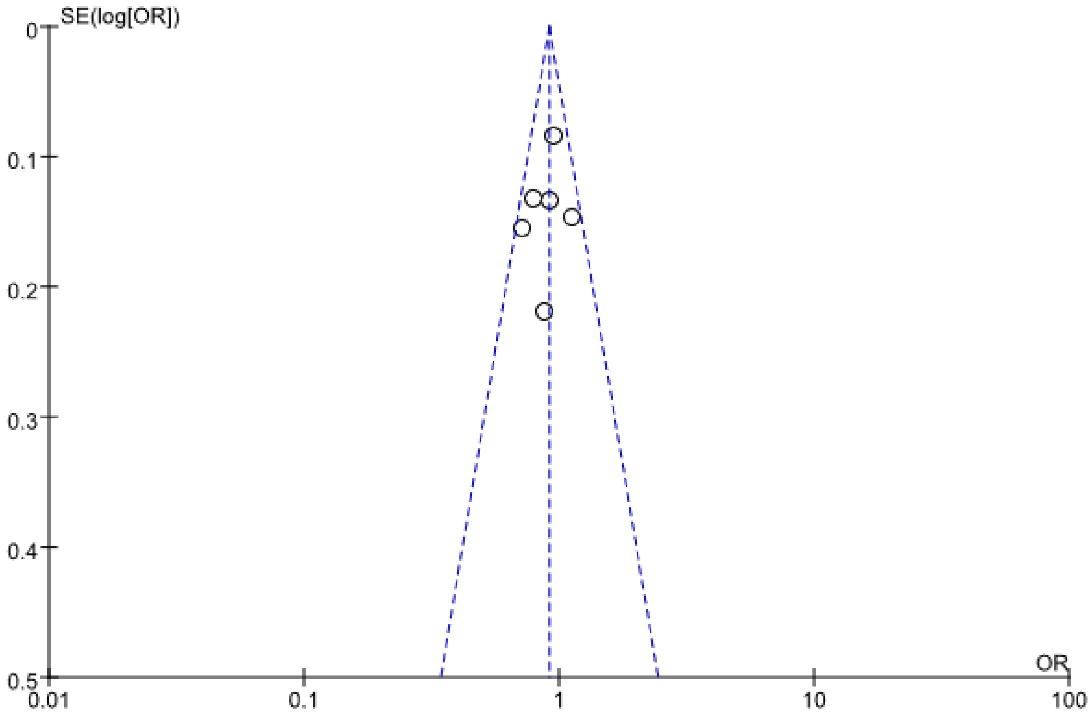

Funnel plot of the **rs10757274** polymorphism and IS under dominant comparison.

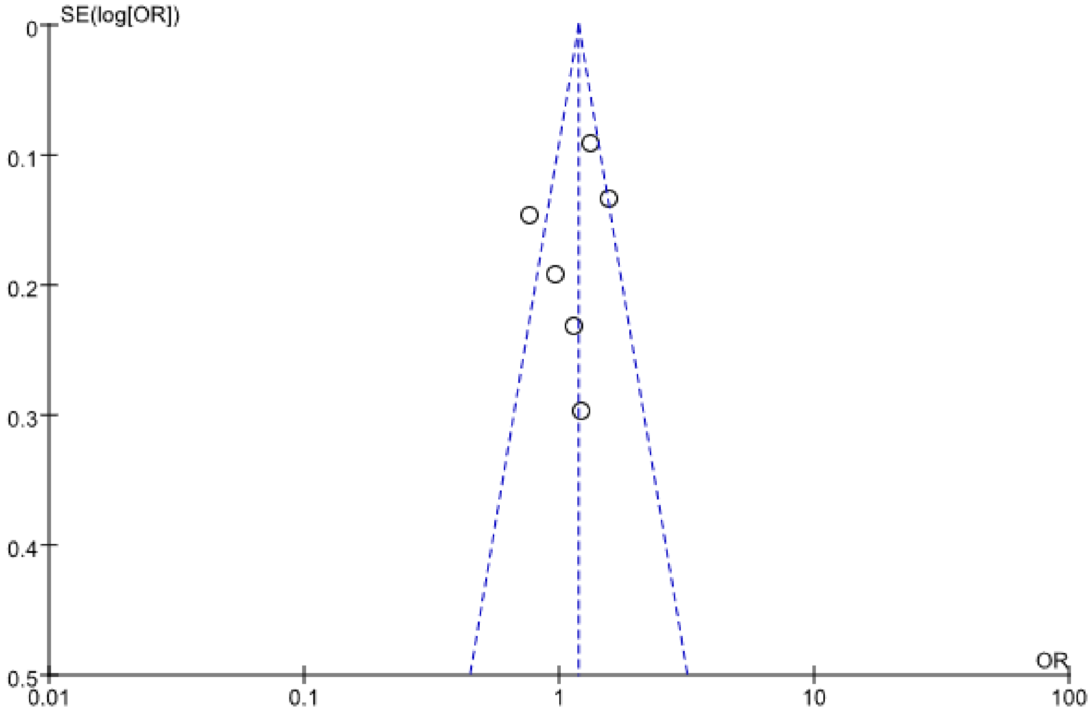

Funnel plot of the **rs10757274** polymorphism and IS under recessive comparison.

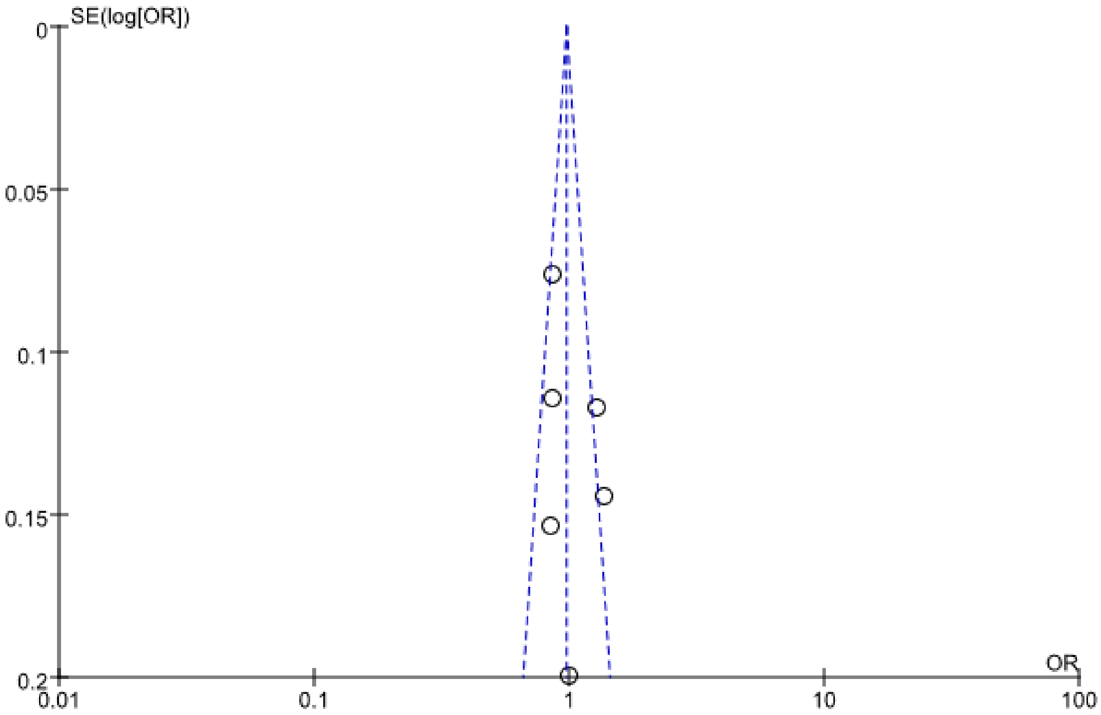

Funnel plot of the **rs10757274** polymorphism and IS under additive comparison.

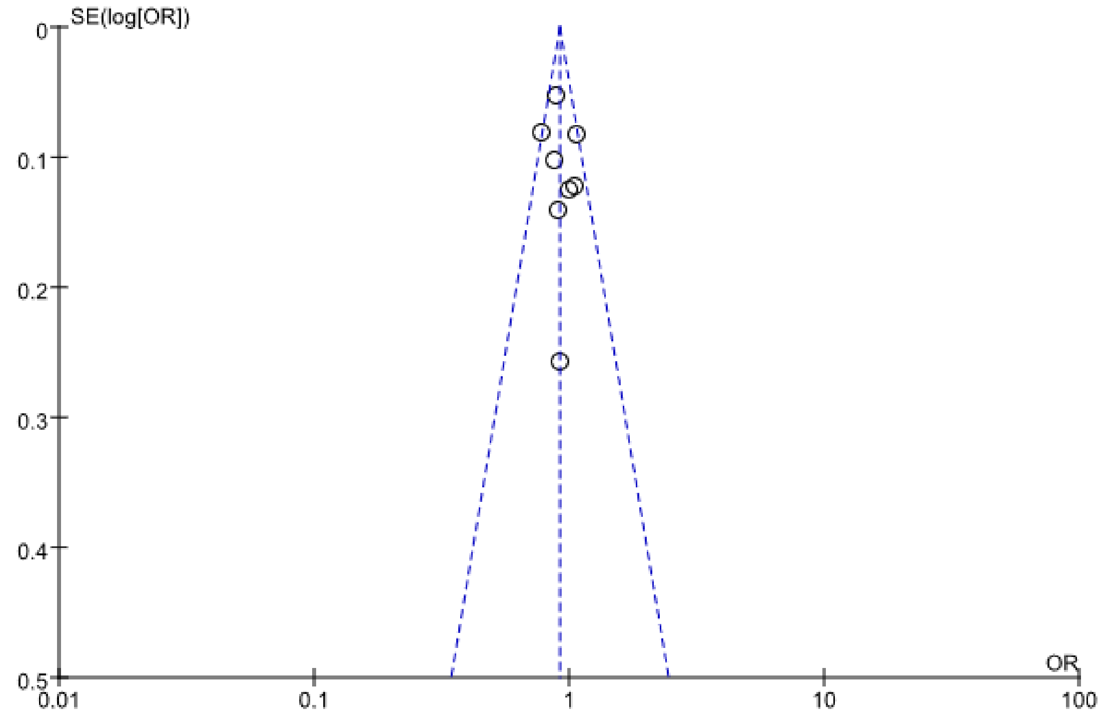

Funnel plot of the **rs10757274** polymorphism and IS under allele comparison.

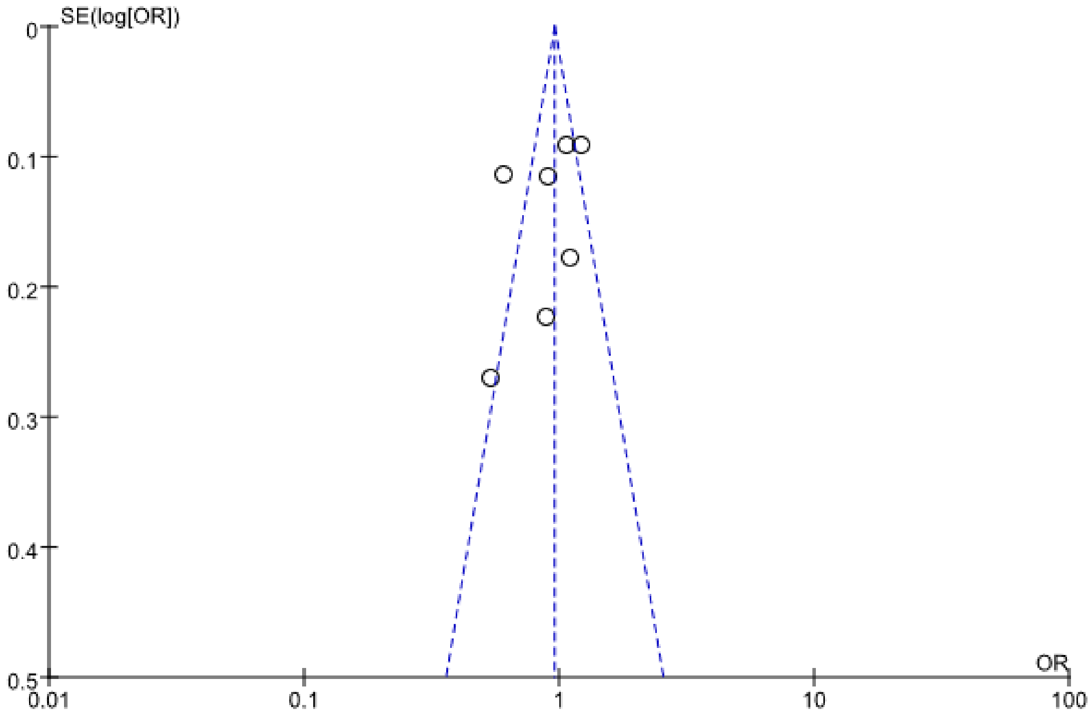

Funnel plot of the **rs10757278** polymorphism and IS under dominant comparison.

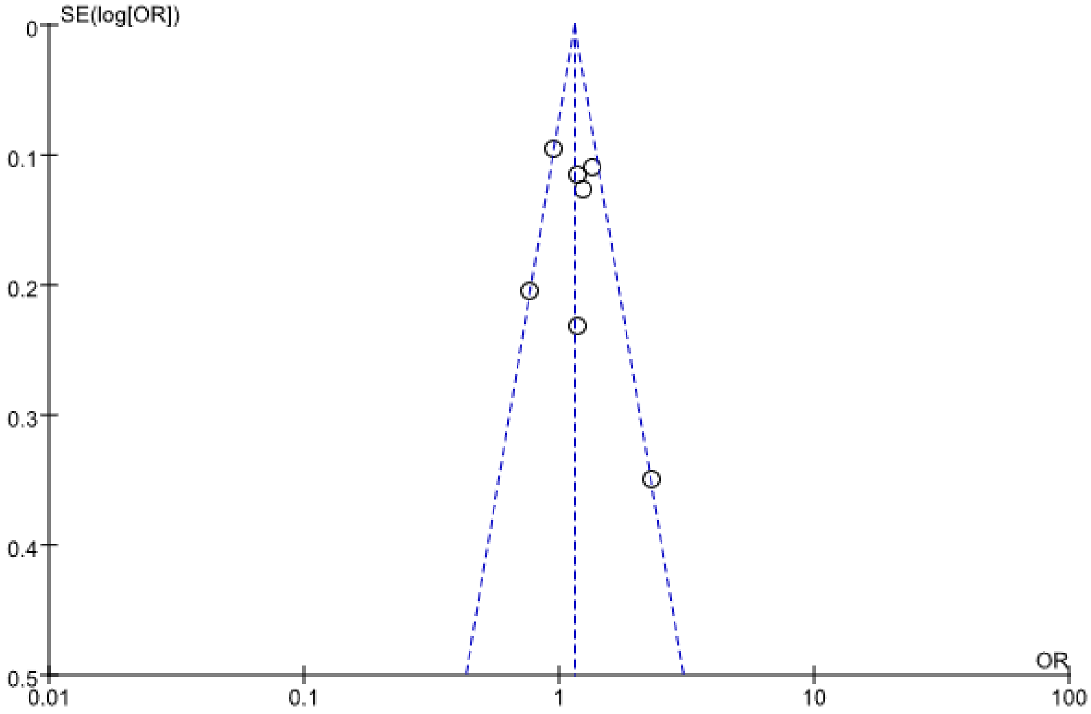

Funnel plot of the **rs10757278** polymorphism and IS under recessive comparison.

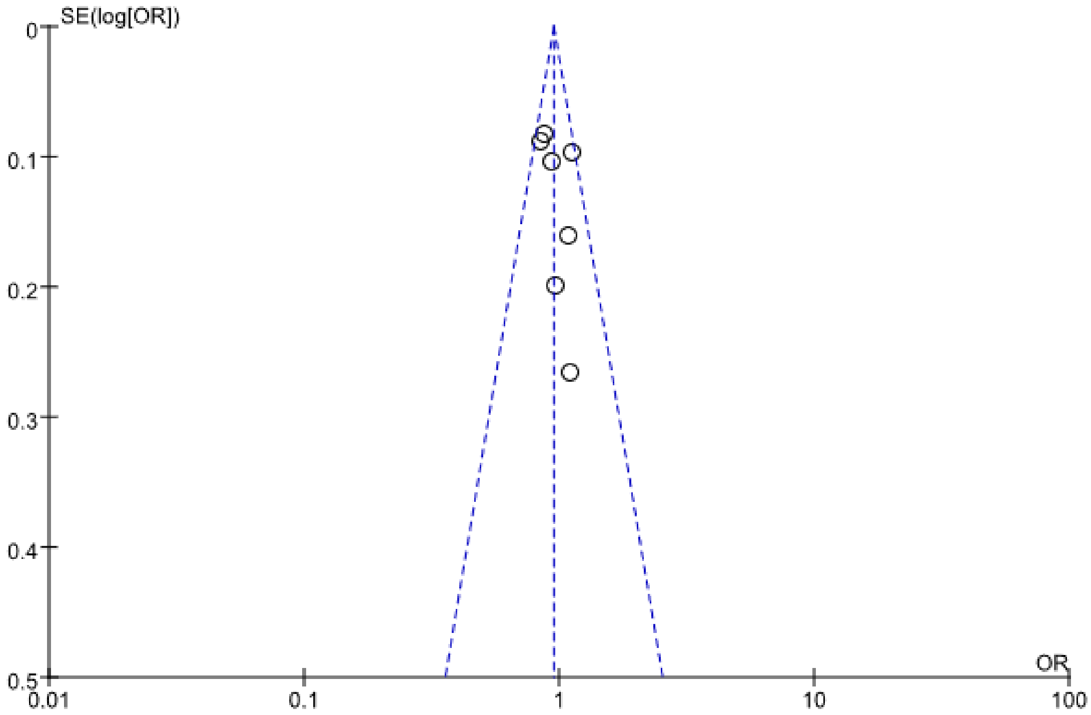

Funnel plot of the **rs10757278** polymorphism and IS under additive comparison.

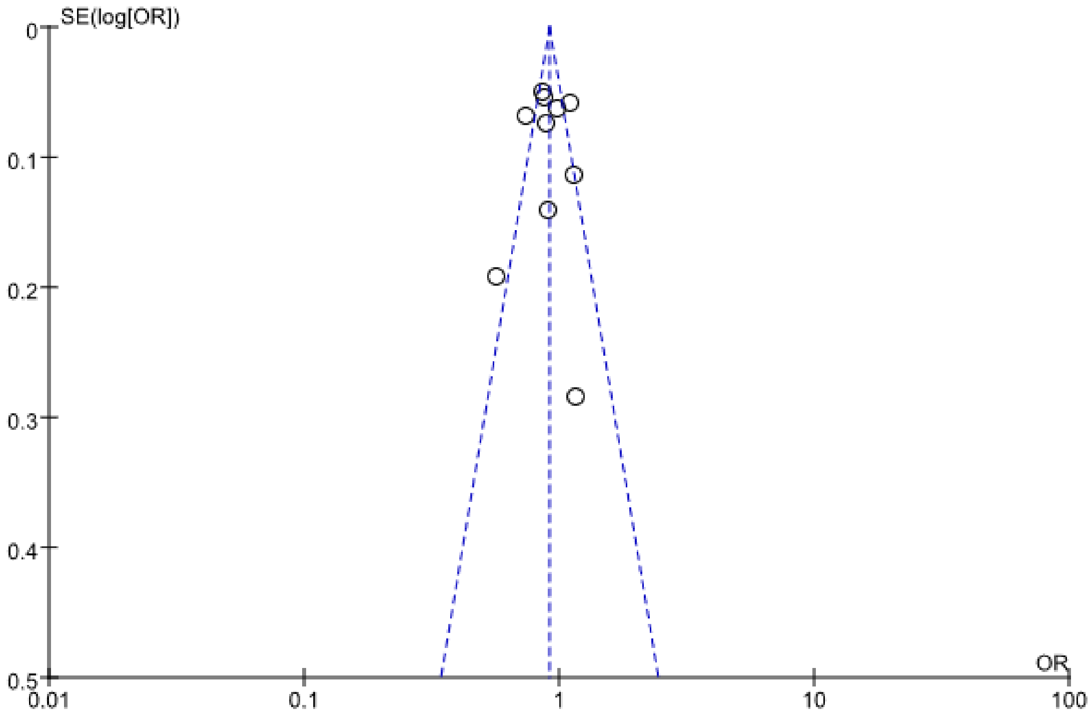

Funnel plot of the **rs10757278** polymorphism and IS under allele comparison.
